# Mapping axon initial segment structure and function by multiplexed proximity biotinylation

**DOI:** 10.1038/s41467-019-13658-5

**Published:** 2020-01-03

**Authors:** Hamdan Hamdan, Brian C. Lim, Tomohiro Torii, Abhijeet Joshi, Matthias Konning, Cameron Smith, Donna J. Palmer, Philip Ng, Christophe Leterrier, Juan A. Oses-Prieto, Alma L. Burlingame, Matthew N. Rasband

**Affiliations:** 1 Department of Neuroscience, Baylor College of Medicine, Houston, TX USA; 2 Department of Molecular and Human Genetics, Baylor College of Medicine, Houston, TX USA; 3 Aix-Marseille Univ, CNRS, INP, NeuroCyto, Marseille, France; 4 Department of Pharmaceutical Chemistry, University of California San Francisco, San Francisco, CA USA; 5 Present Address: Department of Physiology, College of Medicine, Alfaisal University, Riyadh, 11533 Saudi Arabia

**Keywords:** Cellular neuroscience, Molecular neuroscience

## Abstract

Axon initial segments (AISs) generate action potentials and regulate the polarized distribution of proteins, lipids, and organelles in neurons. While the mechanisms of AIS Na^+^ and K^+^ channel clustering are understood, the molecular mechanisms that stabilize the AIS and control neuronal polarity remain obscure. Here, we use proximity biotinylation and mass spectrometry to identify the AIS proteome. We target the biotin-ligase BirA* to the AIS by generating fusion proteins of BirA* with NF186, Ndel1, and Trim46; these chimeras map the molecular organization of AIS intracellular membrane, cytosolic, and microtubule compartments. Our experiments reveal a diverse set of biotinylated proteins not previously reported at the AIS. We show many are located at the AIS, interact with known AIS proteins, and their loss disrupts AIS structure and function. Our results provide conceptual insights and a resource for AIS molecular organization, the mechanisms of AIS stability, and polarized trafficking in neurons.

## Abstract (web summary)

The axon initial segment (AIS) is a specialized location in neurons with clustered ion channels which regulates the distribution of neuronal materials. Here, the authors use proximity biotinylation to examine the AIS proteome and better define its molecular organization.

## Introduction

Axon initial segments (AISs) are located at the interface between somatodendritic and axonal domains of neurons. They have two main functions: to cluster and maintain ion channels in high densities for efficient action potential initiation^1^, and to control neuronal polarity by regulating the differential distribution and trafficking of proteins, vesicles, organelles, and even lipids between axonal and somatodendritic compartments^2–4^. These properties depend on the cytoskeletal and scaffolding proteins AnkyrinG (AnkG) and β4 spectrin. For example, loss of AnkG blocks clustering of Na^+^ and K^+^ channels^5,6^, and AnkG-deficient axons acquire dendritic features, including spines and post-synaptic densities^7^. Pathogenic variants of *ANK3* and *SPTBN4* (the genes encoding AnkG and β4 spectrin proteins in humans, respectively) lead to severe intellectual disability and neuropathy^8,9^, while injuries and diseases may be accompanied by rapid calpain-dependent proteolysis of AnkG and β4 spectrin^10,11^. Recent studies have yielded exciting new insights into AIS physiology, the molecular organization of the AIS, and AIS-regulated protein trafficking^12,13^. Since AIS Na^+^ and K^+^ channel clustering depends on their AnkG-binding motifs^14,15^, it is easy to understand how loss of AnkG disrupts channel clustering. In contrast, the differential trafficking of somatodendritic and axonal proteins is controlled through microtubule- and actin-dependent mechanisms, but the details remain obscure^16–18^.

The gaps in our knowledge of AIS structure and function reflect the paucity of known AIS proteins. Major conceptual advances in AIS structure and function usually follow the identification of new AIS proteins. For example, the recent identification of Trim46 as an AIS-associated microtubule cross-linking factor^19^, and Ndel1 as a regulator of vesicular trafficking at the AIS^20^, yielded key conceptual insights into AIS structure and function. Although ion channels, cytoskeletal scaffolds, and cell adhesion molecules have been reported at the AIS, these likely represent only a fraction of the proteins required for AIS function and structure. Most studies of AIS proteins have focused on those that are enriched at the AIS. However, many proteins may function at the AIS, but unlike AnkG or β4 spectrin, are not restricted to the AIS. For example, although α2 spectrin is in both axons and dendrites, a subset of α2 spectrin forms a detergent-resistant periodic cytoskeleton at the AIS together with AnkG and β4 spectrin^21^. Thus, a major challenge is to identify proteins that participate in AIS function, but that are not exclusively located or enriched at the AIS. In addition, a major experimental limitation of working with AIS proteins is their detergent insolubility due to their association with the AnkG-dependent AIS cytoskeleton; this renders them refractory to purification by conventional immunoaffinity approaches.

We report here the use of proximity biotinylation to overcome the unique experimental challenges of the AIS. We describe a multiplexed strategy that revealed AIS proteins. For some, we describe previously unreported functions. We propose this spatially segregated AIS proteome will be a valuable resource for further study of AIS components, and that this proteome will help overcome the current bottleneck to understanding AIS structure and function.

## Results

### Targeting BirA* to the AIS

**Fig. 1:**
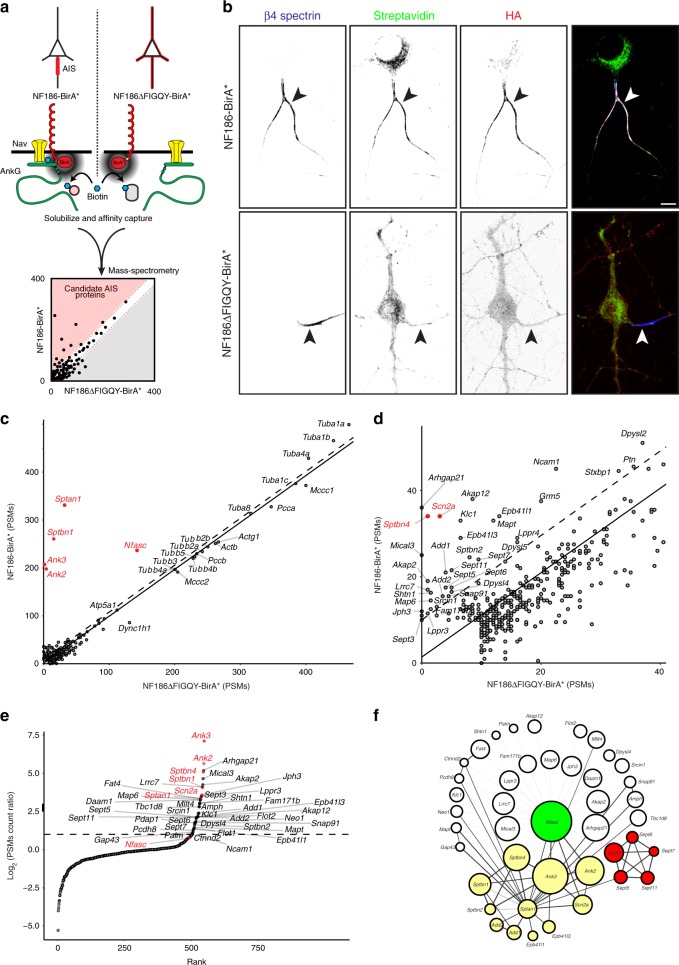
Proximity biotinylation using NF186-BirA* reveals AIS-associated proteins. **a** The experimental strategy using BirA*-dependent proximity biotinylation to identify AIS proteins. **b** Immunolabeling of DIV14 hippocampal neurons transduced at DIV11 using adenovirus to express HA-tagged NF186-BirA* and NF186∆FIGQY-BirA*. From DIV13, neuronal culture media included 50 μM biotin. AIS (arrowheads) are labeled using antibodies against β4 spectrin (blue). The NF186-BirA* and NF186∆FIGQY-BirA* chimeras are labeled using anti-HA antibodies (red). Biotinylated proteins were detected using Alexa488-conjugated Streptavidin. Scale bar = 5 μm. **c**, **d** The number of peptide spectral matches (PSMs) for each biotinylated protein identified by mass spectrometry and shown at different scales. Known AIS and NF186-interacting proteins are indicated in red. The solid line is the least-squares fit of the biotinylated proteins while the dotted lines parallel to the least-squares fit line represent an arbitrary minimum of ten PSMs confidence interval. PSMs are the average PSM counts for two independent replicates for each chimera. **e** Rank plot showing the enrichment of NF186-BirA* PSMs over NF186∆FIGQY-BirA* PSMs. **f** Protein interaction network generated using Cytoscape and the STRING database of protein interactions. With the exception of Nfasc, the size of each circle is proportional to the ratio of the PSMs count for each protein indicated. White circles include proteins with no previously reported function at the AIS. Yellow circles correspond to the ankyrin/spectrin network, and red circles correspond to the septin network.

To identify the AIS proteome we used BirA*-dependent proximity biotinylation^22^. We directed the promiscuous biotin-ligase BirA* to the AIS by fusing it to hemagglutinin (HA)-tagged neurofascin-186 (NF186; Fig. 1a); NF186 is a transmembrane cell adhesion molecule highly enriched at the AIS. BirA* catalyzes the addition of biotin to lysine residues with an effective range of ~10 nm^23^. Thus, proteins within 10 nm of NF186-BirA* will be biotinylated. The high affinity between biotin and streptavidin permits stringent solubilization conditions to purify proteins that are strongly associated with the cytoskeleton; biotinylated proteins can be purified using streptavidin affinity capture, and then identified using mass spectrometry (Fig. 1a). NF186 is targeted to the AIS by its interaction with AnkG. The presence of endogenously biotinylated proteins^24^ and the promiscuity of the BirA* ligase means that appropriate controls are essential. Therefore, we constructed a mutant NF186-BirA* chimera by deleting the cytoplasmic five amino acid sequence (FIGQY) that mediates its interaction with AnkG (Fig. 1a)^25^. Since NF186∆FIGQY-BirA* does not localize to the AIS, biotinylated proteins can be compared to those identified using NF186-BirA*; proteins that are more abundant in the NF186-BirA* purification are candidate AIS proteins (Fig. 1a).

We packaged the large NF186-BirA* and NF186∆FIGQY-BirA* constructs in adenovirus for transduction of hippocampal neurons in vitro. Importantly, the expression of these chimeras was driven by the human neuron-specific enolase (NSE) promoter, since stronger expression using CAG, CMV, EF1α, or hSyn promoters overwhelmed the targeting and clustering machinery resulting in mislocalized proteins. We infected hippocampal neurons at DIV11, and found that by DIV14, NF186-BirA* was enriched at the AIS where it colocalized with β4 spectrin (Fig. 1b). In contrast, NF186∆FIGQY-BirA* was not restricted to the AIS and instead distributed throughout the cell (Fig. 1b). At DIV13, we added 50 μM biotin to the neuron culture medium. One day later (DIV14), the AIS of NF186-BirA*-expressing neurons were strongly labeled by streptavidin, but NF186∆FIGQY-BirA*-expressing neurons had diffuse streptavidin labeling (Fig. 1b). Biotinylated proteins were detected in the soma cytoplasm, but did not strongly colocalize with HA immunostaining, suggesting that this represents endogenously biotinylated proteins. Thus, AIS-localized NF186-BirA* chimeras efficiently biotinylate AIS proteins.

### Mining the NF186 proximity proteome

To identify biotinylated AIS proteins, we solubilized DIV14 transduced and biotin-treated neurons, captured biotinylated proteins using streptavidin coated beads, then identified captured proteins using mass spectrometry. For each protein, we plotted the number of peptide spectral matches (PSMs) as a measure of its enrichment and proximity to NF186-BirA* (Fig. 1c, d). For simplicity, each protein is indicated by its gene name; thus, AnkG is *Ank3* and β4 spectrin is *Sptbn4*. Proteins found at the AIS should have more PSMs present in the NF186-BirA* samples compared to proteins purified from NF186∆FIGQY-BirA* expressing neurons (Fig. 1c, d). Proteins that are non-specifically or intrinsically biotinylated (e.g., carboxylases *Mccc1* and *Pcca*^24^), should have approximately equal numbers of PSMs in both NF186-BirA* and NF186∆FIGQY-BirA* samples. These proteins reside along the diagonal least-squares fit of all purified proteins (Fig. 1c, d, solid diagonal lines). To reduce false positives, we used a stringent cutoff and only investigated proteins biotinylated by NF186-BirA* with at least ten PSMs, and that were above a line parallel to the least-squares fit (Fig. 1c, d, dashed line); this does not mean that proteins below a cutoff of 10 are not AIS proteins, and a less stringent cutoff may yield more candidates. Thus, proteins found above the dashed line in Fig. 1c, d are candidate AIS proteins (Fig. 1d is a magnified view of Fig. 1c). We found the previously described AIS proteins AnkG (*Ank3*), α2 spectrin (*Sptan1*), NF186 (*Nfasc*), β4 spectrin (*Sptbn4*), and Nav1.2 (*Scn2a*; Fig. 1c, d, red) were enriched in the NF186-BirA* expressing neurons compared to NF186∆FIGQY-BirA* expressing neurons. We also found AnkB (*Ank2*) and β2 spectrin (*Sptbn1*) were enriched in the NF186-BirA* samples (Fig. 1c, red), since NF186 is found at low densities in distal axons where it interacts with a cytoskeletal complex consisting of AnkB and β2 spectrin^26,27^, and β2 spectrin was reported at the AIS^28^. We also ranked the biotinylated proteins with PSM counts in the NF186-BirA*-expressing neurons at least twofold above that of NF186∆FIGQY-BirA* expressing neurons (Fig. 1e). The greatest enrichment for proteins in proximity to NF186 are AnkG (*Ank3*) and AnkB (*Ank2*); this is consistent with our strategy to use the non-ankyrin binding NF186∆FIGQY-BirA* chimera as a control. Altogether, these observations demonstrate the robustness of our approach using proximity biotinylation to capture AIS proteins.

Using the proteins enriched in the NF186-BirA* sample (Fig. 1e), we generated a protein interaction network using Cytoscape and the STRING database of protein–protein association networks^29,30^. This network revealed a module with previously known AIS functions (Ankyrins and Spectrins, yellow), a module not previously described (Septins, red), and many proteins with no previously reported connection to NF186 or AIS (Fig. 1f, white).

**Fig. 2:**
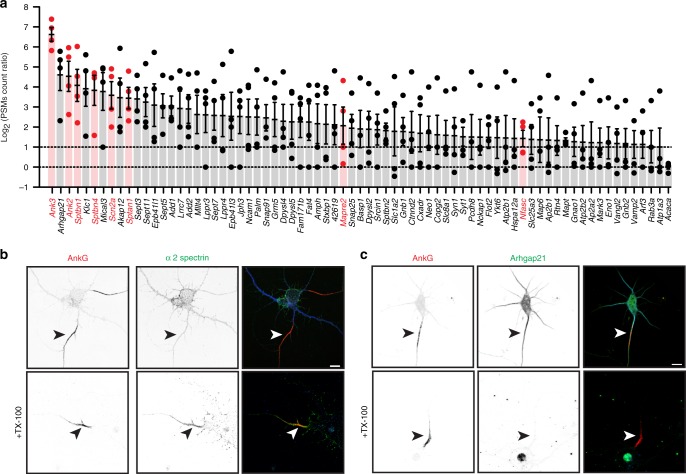
Candidate AIS proteins and their sensitivity to detergent extraction. **a** The ratio (Log_2_) of PSMs in NF186-BirA*-expressing neurons to PSMs in NF186∆FIGQY-BirA*-expressing neurons. Known AIS proteins are shown in red. An endogenously biotinylated carboxylase (*Acaca*), that is equally biotinylated in both NF186-BirA* expressing neurons and NF186∆FIGQY-BirA* expressing neurons, is shown for reference. Dashed lines at 1 indicate twofold enrichment, or equal levels of biotinylation at 0. *n* = 4 independent experiments. Error bars, ±SEM. **b** Immunostaining of DIV14 hippocampal neurons using antibodies against AnkG (red), α2 spectrin (green), and Map2 (blue). The lower panels show the Triton X-100 detergent-resistant pool of α2 spectrin at the AIS. AIS are indicated by arrowheads. Scale bar = 5 μm. **c** Immunostaining of DIV14 hippocampal neurons using antibodies against AnkG (red), Arhgap21 (green) and Map2 (blue). The lower panels show Arhgap21 is solubilized by Triton X-100. AIS are indicated by arrowheads. Scale bar = 10 μm.

To confirm potential AIS proteins, we combined results from four independent experiments (Supplementary Data 1). We identified all PSM count ratios > 2 between NF186-BirA* expressing neurons and NF186∆FIGQY-BirA* expressing neurons (Fig. 2a). A search of the literature revealed that among the 73 proteins preferentially biotinylated in NF186-BirA* expressing neurons, 33% are scaffolding/cytoskeletal proteins and 14% participate in vesicle trafficking.

Since we identified so many candidate AIS proteins, we focused on those that were most enriched (Figs. 1e and 2a), comprises a module (Septins), and had available antibodies. To confirm AIS proteins, we used three criteria: (1) the protein is located at the AIS, (2) the candidate interacts with known AIS proteins, and (3) loss of the candidate perturbs AIS structure or function. We do not suggest that AIS proteins must have any or all of these characteristics, but to find AIS proteins and demonstrate the utility of our approach, these criteria are robust and stringent. Below, we describe examples of proteins that satisfy these criteria and that provide conceptual insights into AIS structure and function.

To determine if a candidate is associated with the AIS, we immunostained DIV12-14 hippocampal neurons. In parallel, we immunolabeled neurons extracted with TX-100 prior to fixation to remove soluble proteins. For example, α2 spectrin (*Sptan1*; Figs. 1c and 2a), is the only neuronal α spectrin and is required for the function of all β-spectrins in somatic, dendritic, and axonal compartments^21^. Thus, α2 spectrin immunoreactivity is found throughout neurons. However, detergent extraction prior to fixation and immunolabeling revealed a pool of insoluble α2 spectrin at the AIS (Fig. 2b, arrowhead) that forms a periodic cytoskeleton with β4 spectrin (*Sptbn4*^21^). In contrast, immunolabeling of Arhgap21 (*Arhgap21*; Figs. 1e and 2a) showed widespread immunoreactivity throughout the neuron, but was soluble in detergent (Fig. 2c).

### Mical3 is a spectrin-interacting AIS protein

**Fig. 3:**
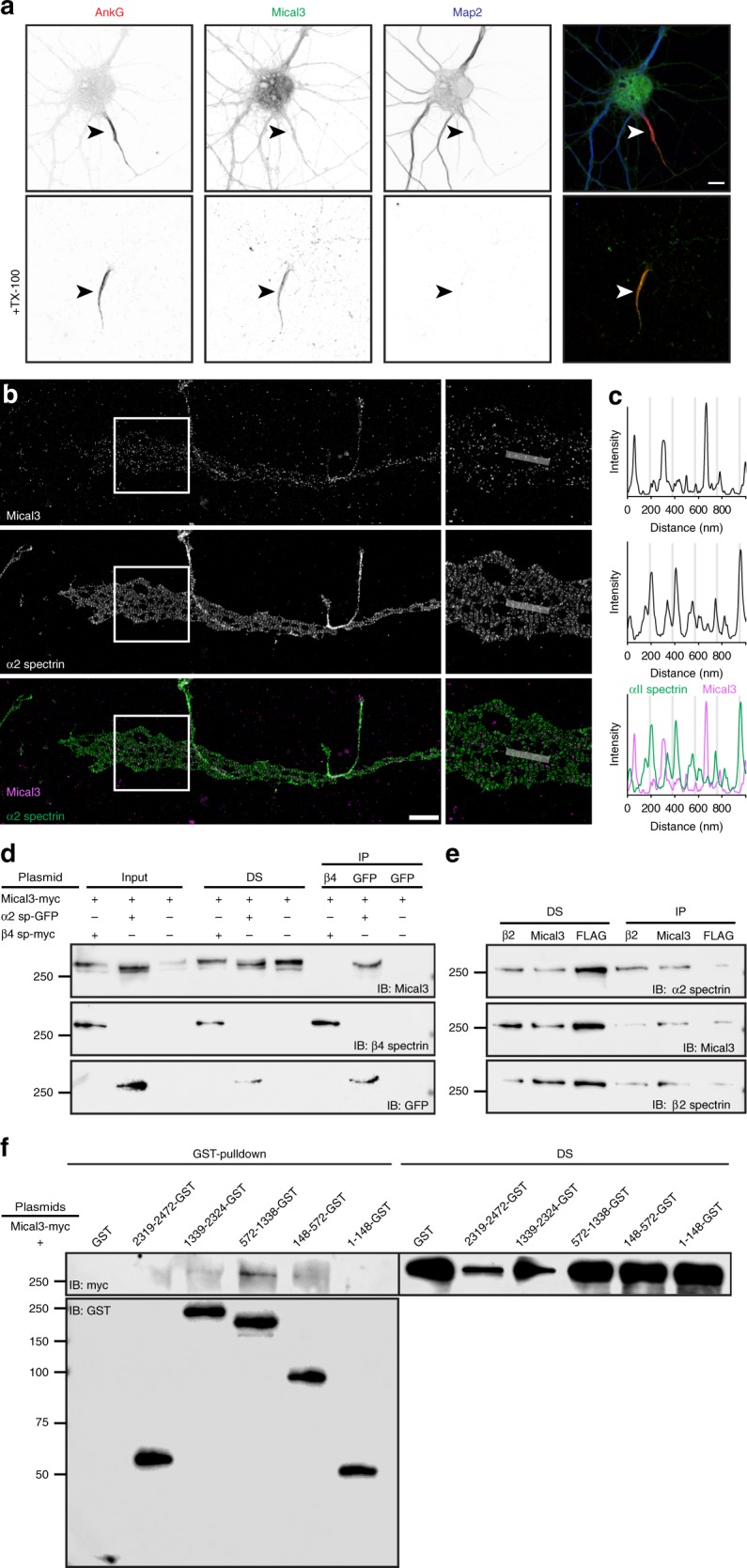
A detergent insoluble pool of spectrin-interacting Mical3 is located at the AIS. **a** Immunostaining of DIV14 hippocampal neurons using antibodies against AnkG (red), Mical3 (green) and Map2 (blue). The lower panels show the Triton X-100 detergent-resistant pool of Mical3 at the AIS. AIS are indicated by arrowheads. Scale bar = 5 μm. **b** DNA-PAINT super-resolution imaging of Mical3 (magenta) and α2 spectrin (green). The boxed regions in the left panels are shown magnified in the right panels. Scale bar = 2 μm. **c** Line scans (gray bars) in the magnified panels in **b** show the normalized fluorescence intensity. Line scans are specific to these images and are not averages across many cells. **d** In vitro co-immunoprecipitation shows Mical3 co-immunoprecipitates with α2 spectrin. DS depleted supernatant, IP immunoprecipitation. **e** α2 spectrin co-immunoprecipitates with Mical3 from mouse brain homogenate. **f** GST-pull-down experiment between Mical3-myc and GST fusion proteins including the amino acids of α2 spectrin indicated. All molecular weight markers are in kilodaltons. Source data are provided as a Source Data file.

The first protein we identified at the AIS was Mical3 (*Mical3*; Figs. 1e and 2a). Mical proteins are oxidoreductases that function to depolymerize F-actin^31^. However, Micals are catalytically inactive since they reside in an auto-inhibited conformation in the absence of stimuli^32^. Immunostaining for Mical3 showed 98% of hippocampal neurons had a detergent-resistant pool at the AIS that colocalized with AnkG (Fig. 3a, arrowhead). We defined the precise distribution of Mical3 in the AIS using super-resolution Single-Molecule Localization Microscopy (SMLM)^33^. To simultaneously visualize the AIS cytoskeleton and Mical3, we used DNA-PAINT imaging, which allows multicolor SMLM^34^. Consistent with previous reports^21^, we found two peaks of α2 spectrin immunoreactivity centered on a periodicity of ~190 nm (Fig. 3b, c). SMLM of AIS Mical3 did not show clear periodicity, but instead showed punctate immunoreactivity that did not overlap with α2 spectrin (Fig. 3b, c). Line scans show that detergent-resistant clusters of Mical3 are usually nested between α2 spectrin.

Does Mical3 interact with AIS proteins? We performed in vitro co-immunoprecipitation experiments between Mical3 and known AIS proteins. Although we did not detect interactions between Mical3 and NF186 or AnkG, we found interactions between Mical3 and AIS α2 spectrin (Fig. 3d). Immunoprecipitation of Mical3 from brain homogenate also co-immunoprecipitated α2 spectrin (Fig. 3e). One recent mass spectrometry-based screen for Mical3-binding partners also reported Mical3’s top interactors in HEK293T cells were α2 spectrin (*Sptan1*) and β2 spectrin (*Sptbn1*)^35^. To define where Mical3 interacts with α2 spectrin, we performed pull-down experiments using GST fusion protein fragments of α2 spectrin. These experiments revealed that Mical3 binds α2 spectrin at or near its SH3 domain (AA 572-1338; Fig. 3f). Remarkably, Mical proteins received their name and were originally identified based on their interaction with the SH3 domain of CasL (Molecule Interacting with CasL)^36^.

**Fig. 4:**
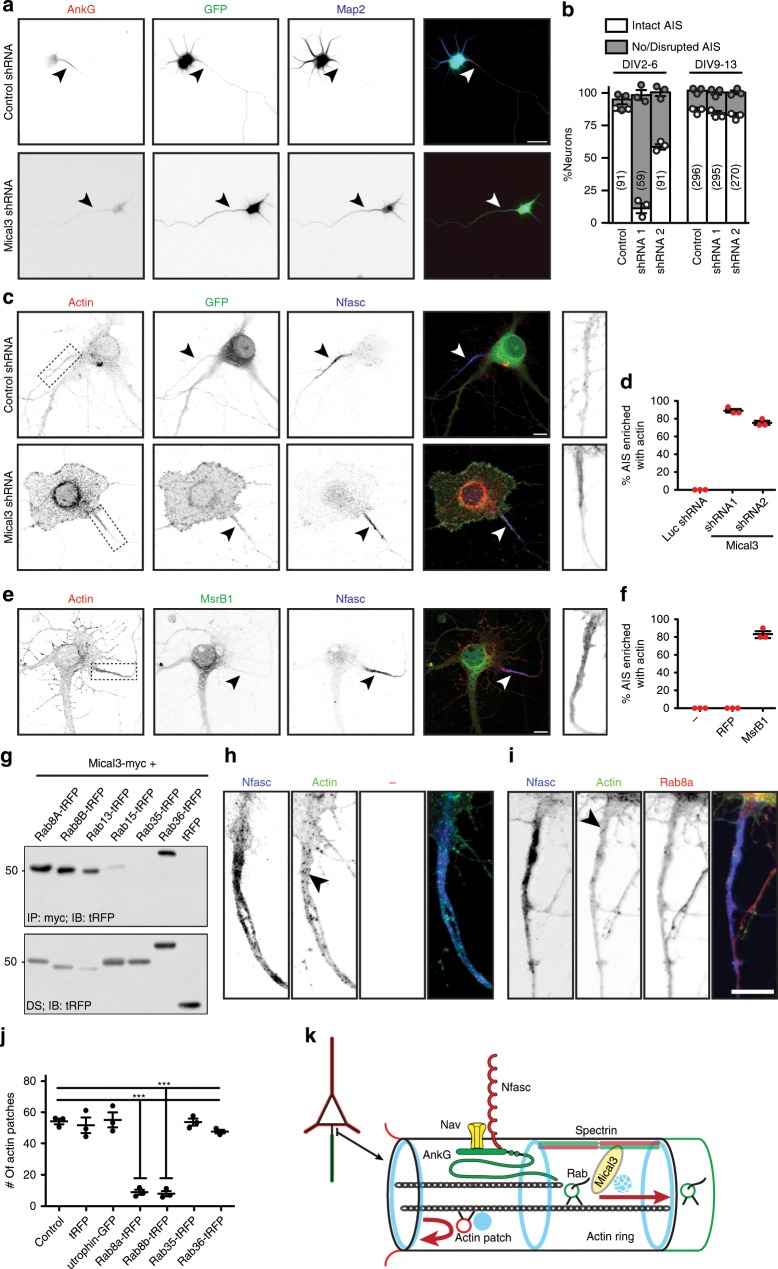
Mical3 regulates AIS assembly and actin patches. **a** Immunostaining of DIV6 hippocampal neurons transfected at DIV2 with pSuper or Mical3 shRNA. Scale bar = 20 µm. **b** Quantification of neurons with intact or no/disrupted AIS after transfection with pSuper control shRNA, or two different Mical3 shRNAs at DIV2-6 or DIV9-13. *n* = 3 independent experiments and total number of neurons counted is shown. Error bars, ±SEM. **c** Immunostaining of DIV13 hippocampal neurons transfected at DIV9 with pSuper or Mical3 shRNA. Scale bar = 10 µm. **d** Quantification of the percentage of neurons transfected with the indicated shRNA that show AIS enriched with actin. *n* = 3 independent experiments/condition with 15 neurons/condition. Error bars, ±SEM. **e** Immunostaining and labeling of hippocampal neurons transfected with MsrB1 cDNA. Phalloidin (red), MsRB1 (green), and Nfasc (blue). Arrowheads indicate the AIS. Scale bar = 10 µm. **f** Quantification of the percent of AIS enriched with actin as indicated by phalloidin labeling in the presence or absence of MsrB1. *n* = 3 independent experiments/condition with 10 neurons/condition. Error bars, ±SEM. **g** In vitro immunoprecipitation of Mical3-myc using myc antibodies co-immunoprecipitates some tRFP tagged RabGTPases. IP immunoprecipitation, IB immunoblotting. Molecular weight markers are in kilodaltons. **h**, **i** Labeling of AIS actin patches (arrowhead) using phalloidin (green) in untransfected DIV 14 neurons (**h**), or Rab8a-tRFP-transfected DIV 14 hippocampal neurons using phalloidin (green) and antibodies against Nfasc (blue) and tRFP (red) (**i**). Actin patches are indicated by the arrowhead. Scale bar = 10 µm. **j** The number of actin patches/AIS in neurons transfected with the indicated plasmid. *n* = 3 independent experiments with 10 neurons/experiment. (*F* = 52.63, df total = 20) ***p = 8.29 × 10^−9^; one-way ANOVA. Error bars, ±SEM. **k** Model for Mical3 function at the AIS. The cartoon illustrates that Rab GTPases on vesicles carrying axonal cargoes activate Mical3, resulting in depolymerization of actin patches. Vesicles carrying somatodendritic cargoes reverse direction at actin patches and do not enter the axon. Source data are provided as a Source Data file.

What does Mical3 do at the AIS? We generated shRNAs to silence Mical3’s expression. To determine if Mical3 is important for AIS assembly, we transfected plasmids at DIV2, and examined AIS at DIV6. Whereas control short-hairpin RNA (shRNA) had no effect on AIS assembly (Fig. 4a, arrowhead), Mical3 shRNA frequently disrupted or blocked AnkG clustering and AIS assembly (Fig. 4a, b). In contrast, transfection of more mature neurons at DIV9 did not disrupt AIS maintenance at DIV13 (Fig. 4b, c). However, silencing expression of Mical3 dramatically altered cell morphology and increased AIS actin (Fig. 4c, box and right, d). Similarly, transfection of the methionine sulfoxide reductase B1 (MsrB1), a Mical3 antagonist^37^, did not disrupt the AIS but instead promoted the accumulation of actin at the AIS (Fig. 4e, f). Thus, reduced Mical3 activity in mature neurons promotes actin accumulation at the AIS.

Although normally auto-inhibited, the enzymatic activity of Mical proteins can be activated by Rab GTPases, and different Rabs can interact with Micals to locally control activity^38,39^. For example, the interaction between Mical3 and Rab8a regulates vesicle exocytosis^40^. We confirmed Mical3 interacts with some Rab GTPases, but not others (Fig. 4g). Dynamic actin patches at the AIS may function as sorting stations to exclude vesicles carrying dendritic cargoes from the axon^17^. To increase Mical3 activity and to determine if Mical3 can regulate AIS actin patches, we counted the number of AIS actin patches in neurons transfected at DIV12 with Mical3-interacting Rab GTPases used to activate Mical3’s oxidoreductase activity. At DIV14, we found AIS actin patches in untransfected neurons (Fig. 4h, arrowhead), but transfection of Mical3-binding Rab8a and Rab8b significantly reduced the number of AIS actin patches (Fig. 4i, j). However, expression of utrophin (actin-binding), Rab35 (Mical3-non-binding), and Rab36 (Mical3-binding) did not affect the number of AIS actin patches. These results suggest AIS Mical3 may be bound to the AIS spectrin cytsokeleton and activated by specific Rab GTPases found on vesicles with axonal cargoes; we speculate activation of Mical3 disassembles AIS actin patches, permitting vesicles containing axonal cargoes to pass through the AIS and enter the axon (Fig. 4k). Those without the appropriate Rab GTPase reverse direction at the actin patch^17^ and are excluded from the axon.

### Septins stabilize and maintain AIS AnkG

**Fig. 5:**
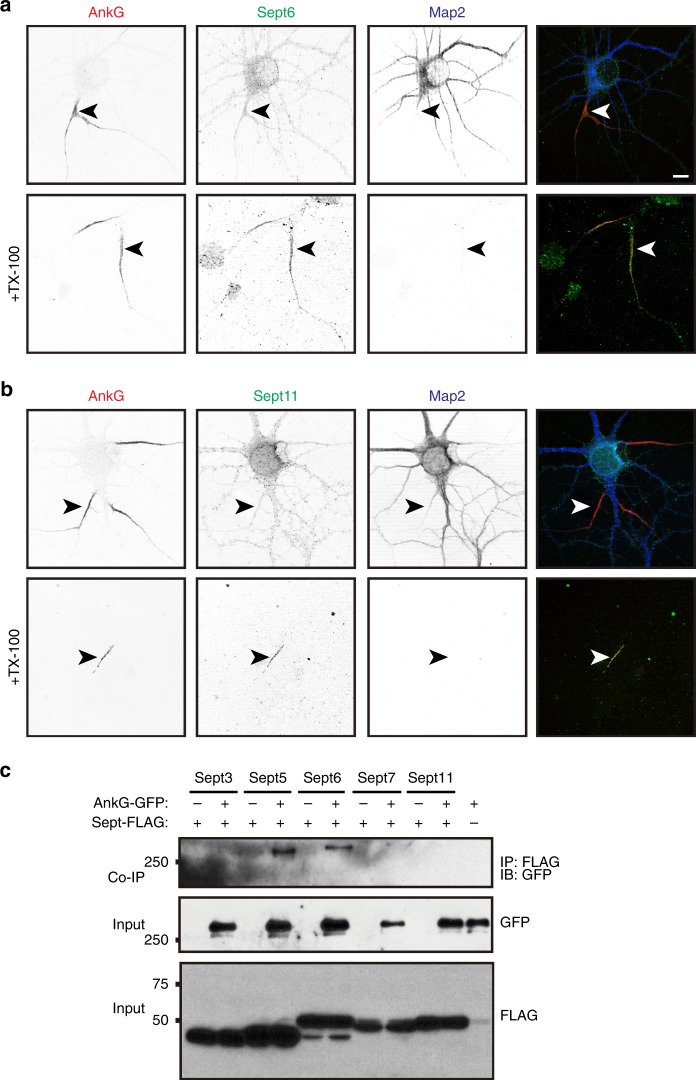
A detergent insoluble pool of AnkG-associated Septins is located at the AIS. **a**, **b** Immunostaining of DIV14 hippocampal neurons using antibodies against AnkG (red), Sept6 (**a**, green), Sept11 (**b**, green) and Map2 (blue). The lower panels show the Triton X-100 detergent-resistant pools of Sept6 (**a**) and Sept11 (**b**) at the AIS. AIS are indicated by arrowheads. Scale bar = 5 μm. **c** In vitro co-immunoprecipitation shows AnkG-GFP co-immunoprecipitates with Sept5-FLAG and Sept6-FLAG. IP immunoprecipitation, IB immunoblotting. Molecular weight markers are in kilodaltons. Source data are provided as a Source Data file.

The NF186-BirA* protein interaction network included a prominent module with 5 different Septins (*Sept3, Sept5, Sept6, Sept7*, and *Sept11*; Figs. 1f and 2a). This attracted our attention since Septins modulate the functions of actin and microtubules, create membrane boundaries, and function as scaffolds—all important properties for the AIS^41^. To determine if Septins are located at the AIS, we immunostained DIV14 hippocampal neurons using antibodies against Septins 3, 5, 6, 7, and 11. All were widely distributed throughout neurons in both axonal and somatodendritic domains (Fig. 5a, b and Supplementary Fig. 1). Detergent extraction prior to fixation revealed a detergent-resistant pool of Septins 5, 6, 7, and 11 that colocalized with AnkG (Fig. 5a, b and Supplementary Fig. 1b, c, arrowheads). Among these Septins, Sept5 and Sept6 can co-immunoprecipitate AnkG (Fig. 5c). Further analysis of Sept5 and 6 showed that 55 and 73% of AIS, respectively, had a detergent-resistant pool of these proteins. Neuronal expression of Sept5 and Sept6 deletion mutants lacking either their N- or C-termini showed that Sept5ΔN had no effect on AIS AnkG, but expression of Sept5ΔC blocked AIS assembly and mislocalized AnkG to somatodendritic regions (Supplementary Fig. 2a). These results suggest AnkG interacts with an N-terminal domain of Sept5. In contrast, neither expression of Sept6ΔN nor Sept6ΔC affected the AIS localization of AnkG (Supplementary Fig. 2b). Nevertheless, expression of Sept6ΔC dramatically altered the membrane organization of the cell by inducing the formation of large membrane sheets in both axonal and somatodendritic domains (Supplementary Fig. 2b).

**Fig. 6:**
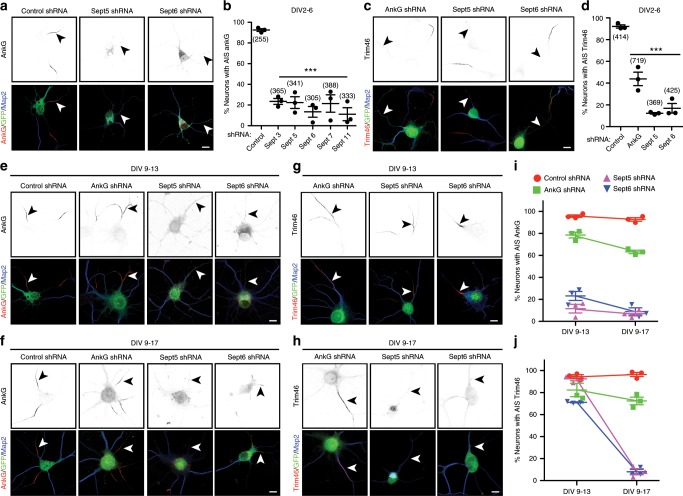
Septins stabilize and maintain AnkG at the AIS. **a**, **c** Immunostaining of DIV6 neurons, transfected at DIV2 with control, Sept5, and Sept6 shRNAs, using antibodies against AnkG (**a**, red) or Trim46 (**c**, red), GFP (green), and Map2 (blue). Arrowheads indicate AIS. In **c** each field also shows AIS of untransfected neurons. **b** Percentage of neurons with AIS AnkG. DIV2 neurons transfected using shRNAs against Luciferase (Luc), and Septins 3, 5, 6, 7, and 11. Neurons were immunostained for AnkG immunoreactivity at DIV6. (*F* = 31.54, df total = 17) ***p = 1.66 × 10^−6^; one-way ANOVA. *n* = 3 independent experiments, total neurons: Luc shRNA = 255, Sept3 shRNA = 365, Sept5 shRNA = 341, Sept6 shRNA = 305, Sept7 shRNA = 388, Sept11 shRNA = 333. **d** Percentage of neurons with AIS Trim46. DIV2 neurons transfected using shRNAs against Luc, AnkG, Sept5, and Sept6. Neurons were immunostained for Trim46 immunoreactivity at DIV6. (*F* = 88.68, df total = 11) ***p = 1.76 × 10^−6^; one-way ANOVA. *n* = 3 independent experiments, total neurons: Luc shRNA = 414, AnkG shRNA = 719, Sept5 shRNA = 369, Sept6 shRNA = 425. **e**, **f** Immunostaining of DIV13 (**e**) and DIV 17 (**f**) neurons, transfected at DIV9 with control, AnkG, Sept5, and Sept6 shRNAs, using antibodies against AnkG (red), GFP (green), and Map2 (blue). Arrowheads indicate AIS. **g**, **h** Immunostaining of DIV13 or DIV17 neurons, transfected at DIV9 with AnkG, Sept5, and Sept6 shRNAs, using antibodies against Trim46 (red), GFP (green), and Map2 (blue). Arrowheads indicate AIS of transfected neurons. **i** Percentage of neurons with AIS AnkG. Neurons transfected at DIV9 using shRNA to Luc, AnkG, Sept5, or Sept6. AIS were labeled for AnkG at DIV13 or DIV17. *n* = 3 independent experiments, total neurons: (DIV9-13) Luc shRNA = 423, AnkG shRNA = 443, Sept5 shRNA = 252, Sept6 shRNA = 455; (DIV9-17) Luc shRNA = 423, AnkG shRNA = 515, Sept5 shRNA = 312, Sept6 shRNA = 245. **j** Percentage of neurons with AIS Trim46. Neurons transfected at DIV9 using shRNA to silence Luc, AnkG, Sept5, or Sept6. AIS were labeled for Trim46 at DIV13 or DIV17. *n* = 3 independent experiments, total neurons: (DIV9-13) Luc shRNA = 454, AnkG shRNA = 544, Sept5 shRNA = 240, Sept6 shRNA = 253; (DIV9-17) Luc shRNA = 256, AnkG shRNA = 390, Sept5 shRNA = 249, Sept6 shRNA = 245. All error bars, ±SEM. All scalebars = 5 µm. Source data are provided as a Source Data file.

To determine the function of AIS Septins, we silenced their expression during early development (from DIV2 to DIV6) and examined AIS AnkG. We found that loss of any AIS Septin blocked AnkG clustering (Fig. 6a, b). Since Septins can regulate microtubules, we examined how loss of Sept5 and Sept6 affects the assembly of bundled AIS microtubules as indicated by AIS Trim46^19^. Whereas silencing of AnkG reduced by ~60% the number of neurons with AIS Trim46, silencing Sept5 and Sept6 caused even more dramatic loss of AIS Trim46 (Fig. 6c, d). Thus, Septins are required for AIS assembly.

AIS AnkG is essential to maintain neuronal polarity^7^. To determine if Septins also contribute to AIS maintenance, we silenced expression of Sept5 and Sept6 in mature neurons since they bind AnkG (Fig. 5c). Silencing AnkG expression from DIV9-13 (Fig. 6e) and DIV9-17 (Fig. 6f) resulted in 22 and 37% reduction in the number of neurons with AIS AnkG, respectively (Fig. 6i); this modest reduction is consistent with AnkG’s very long half-life^7^. In contrast, silencing Sept5 and Sept6 expression resulted in rapid and nearly complete loss of AIS AnkG in both DIV9-13 (Fig. 6e, i) and DIV9-17 neurons (Fig. 6f, i). When we examined AIS Trim46 in neurons transfected with AnkG shRNA, we found AIS Trim46 was largely preserved in both DIV9-13 (Fig. 6g, j) and DIV9-17 neurons lacking AnkG (Fig. 6h, j). Remarkably, in contrast to loss of AIS AnkG, silencing Sept5 and Sept6 did not affect AIS Trim46 in DIV9-13 neurons (Fig. 6g, j); the effect of Septin loss on Trim46 takes longer than the effect on AnkG, and Trim46 is lost from the AIS only in DIV9-17 neurons (Fig. 6h, j). The concurrent retention of AIS Trim46 and loss of AnkG after Septin knockdown, with loss of Trim46 much later than AnkG, reveals that AIS Septins function to directly stabilize and maintain AIS AnkG in neurons. Thus, proximity biotinylation using NF186-BirA* reveals AIS proteins, leading to conceptual insights for AIS structure and function.

### Ndel1 proximity proteome maps the shallow AIS cytoplasm

**Fig. 7:**
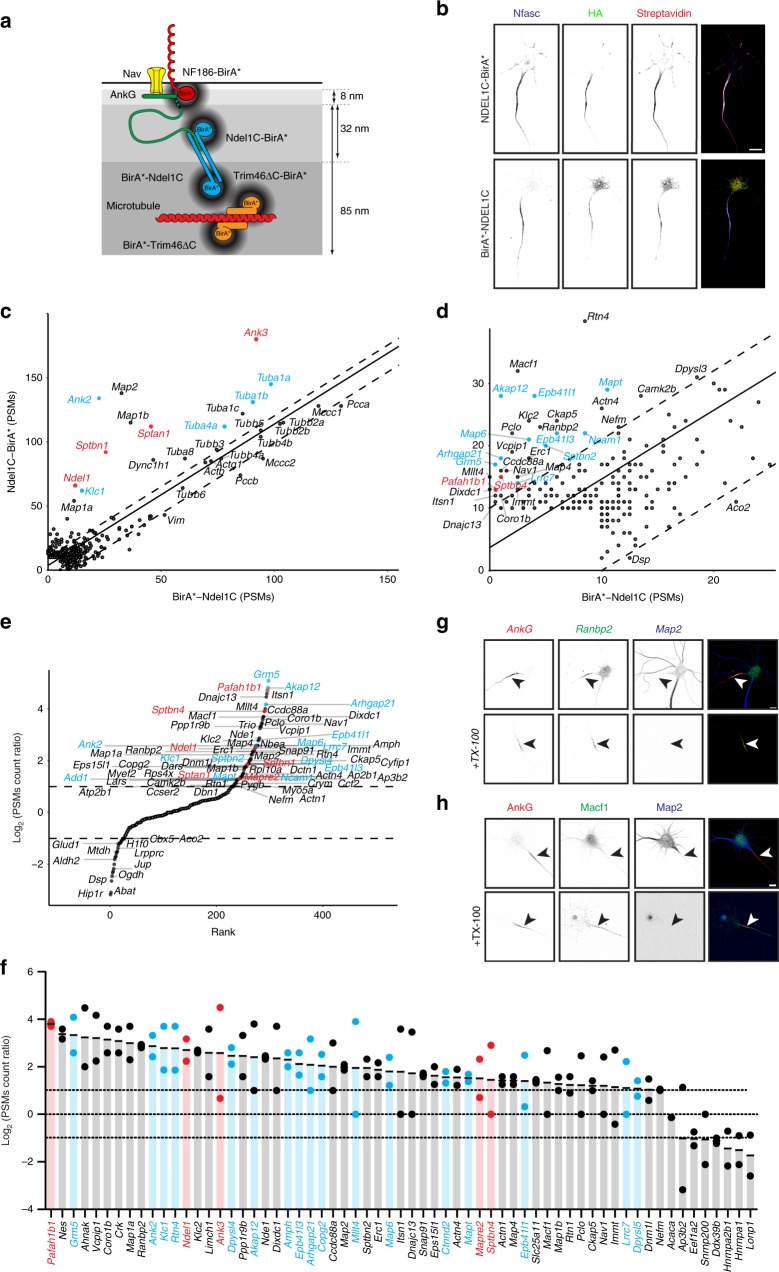
The Ndel1 proximity proteome. **a** Cartoon of BirA*-containing chimeras used to identify AIS proteins and their locations relative to β4 spectrin (not shown). **b** Immunolabeling of DIV14 hippocampal neurons transduced at DIV12 using adenovirus to express HA-tagged Ndel1C-BirA* and BirA*-Ndel1C. AIS are labeled using antibodies against Neurofascin (Nfasc, blue). The Ndel1C-BirA* and BirA*-Ndel1C chimeras are labeled using anti-HA antibodies (green). Biotinylated proteins were detected using Alexa594-conjugated Streptavidin. Scale bar = 5 μm. **c**, **d** The number of PSMs for each protein biotinylated by Ndel1C-BirA* and BirA*-Ndel1C, and identified by mass spectrometry; panels **c** and **d** are shown at different scales. Known AIS proteins are indicated in red. Proteins enriched and previously identified as candidate AIS proteins in the NF186-BirA* samples are indicated in blue. The solid line is the least-squares fit of the biotinylated proteins while the dotted lines parallel to the least-squares fit line represent an arbitrary minimum of ten PSMs confidence interval. Peptide counts for BirA*-Ndel1C are the average of two replicates, while results for Ndel1C-BirA* are from one experiment. **e** Rank plot showing the enrichment of PSMs for a given protein in the Ndel1C-BirA* samples over the PSMs for a given protein found in the BirA*-Ndel1C samples. Known AIS proteins are indicated in red. Proteins enriched and previously identified as candidate AIS proteins in the NF186-BirA* samples are indicated in blue. **f** The average ratio (Log_2_) of PSMs in Ndel1C-BirA* to PSMs in BirA*-Ndel1C expressing neurons. Known AIS proteins shown in red. Proteins also identified using NF186-BirA* chimeras shown in blue. Dashed lines indicate twofold enrichment, or equal levels of biotinylation. *n* = 2 independent experiments. **g** Immunostaining of DIV14 cultured hippocampal neurons using antibodies against AnkG (red), Ranbp2 (green), and Map2 (blue). The lower panels show detergent-resistant Ranbp2 at the AIS. AIS are indicated by arrowheads. Scale bar = 5 µm. **h** Immunostaining of DIV14 cultured hippocampal neurons using antibodies against AnkG (red), Macf1 (green), and Map2 (blue). The lower panels show the detergent-resistant Macf1 at the AIS. Arrowheads indicate. Scale bar = 5 µm.

Three-dimensional (3D) STORM imaging previously showed that NF186 and Nav channel interactions with AnkG occur about 8 nm above β4 spectrin (Fig. 7a)^42^. Therefore, AIS proteins found deeper in the AIS cytoplasm might be missed using a membrane-associated BirA* chimera since its range is only ~10 nm. Therefore, we used the AIS cytoplasmic protein Ndel1^20^ to map the deeper molecular structure of the AIS (Fig. 7a). The C-terminal half of Ndel1 (Ndel1C; amino acids 166 to 345) binds to the C-terminus of AnkG (~32 nm below the AIS membrane)^42^ and mediates its localization to the AIS^20^. Unfortunately, the precise amino acids responsible for Ndel1’s AIS targeting are unknown; thus, we could not use a control chimera like the non-localizing NF186∆FIGQY-BirA*. In a pilot experiment we compared proteins biotinylated by BirA* alone to the pool of endogenously biotinylated proteins. BirA* alone has been used as a control for biotinylation in vivo^43^. We found that after adenovirus-mediated delivery of BirA*, the biotin-ligase alone is found throughout the neuron and biotinylates tubulins (Supplementary Fig. 3). Nevertheless, we were concerned that BirA* alone might be a poor control for chimeras specifically targeted to distinct domains or compartments (AIS, membrane, etc.). As an alternative strategy to reveal proteins in the vicinity of AIS Ndel1, we constructed two chimeras consisting of the HA-tagged Ndel1C AnkG-binding domain and BirA* linked to the N- (BirA*-Ndel1C) or C-terminus (Ndel1C-BirA*). Since the N- and C-terminal BirA* are separated by a largely unstructured 179 amino acids^44^, the contour length of Ndel1C (3.4–4.0 Å/residue)^45^ suggests the two chimeras may map regions as much as ~60–70 nm apart. Thus, by comparing these chimeras we can spatially map the AIS cytoplasm. We packaged these in adenoviruses, transduced DIV11 neurons, and found that by DIV14 the chimeras were enriched at the AIS and colocalized with NF186 (*Nfasc*). Addition of biotin at DIV13 resulted in biotinylation of AIS proteins by DIV14 (Fig. 7b); we purified the biotinylated proteins and performed mass spectrometry. We then compared proteins biotinylated by the Ndel1C-BirA* chimera, to proteins biotinlyated by the BirA*-Ndel1C chimera (Fig. 7c–e). We found that Ndel1C-BirA* biotinylated its binding partner Lis1 (*Pafah1b1*; also located at the AIS^20^) and AnkG (*Ank3*) more efficiently than the Ndel1C-BirA* chimera (Fig. 7d, e), suggesting that it is oriented with the BirA* towards the membrane and AnkG (Fig. 7a).

To further confirm AIS proteins, we combined results from two independent experiments (Supplemenary Data 1). We identified all PSM count ratios >2 between Ndel1C-BirA* and BirA*-Ndel1C chimeras (Fig. 7f). We found that 20/55 proteins preferentially biotinylated by the Ndel1C-BirA* chimera were common to those biotinylated by NF186-BirA* (Fig. 7f, blue). For example, Map6 and Klc1 were identified and enriched in the Ndel1C-BirA* biotinylated proteins >2-fold over BirA*-Ndel1C biotinylated proteins (Fig. 7f). Map6 was previously reported in axons and shown to stabilize microtubules^46^, while Klc1 participates with kinesin heavy chains to transport cargoes along microtubules. Importantly, antibodies against Map6 and Klc1 label detergent insoluble AIS pools (98% and 88% of AIS, respectively; Supplementary Fig. 4a, e). Both interact with AnkG (Supplementary Fig. 4b, f), and silencing of each using shRNA disrupted or blocked AIS assembly to varying degrees (Supplementary Fig. 4c, d, g, and h). Thus, two independent proximity biotinylation experiments using unique AIS-targeted chimeras converge on many of the same proteins, strongly suggesting they are AIS proteins. The range of the BirA* also implies that proteins biotinylated by Ndel1C-BirA* and NF186-BirA* are located in a shallow region of the AIS cytoplasm that is spanned by AnkG (Fig. 7a).

In addition to proteins preferentially biotinylated by both Ndel1C-BirA* and NF186-BirA* (Fig. 7c–f, blue), we also found proteins that were preferentially biotinylated by Ndel1C-BirA* but not NF186-BirA*, suggesting that they reside deeper in the AIS cytoplasm. For example, immunolabeling of Ranbp2 (*Ranbp2*) showed it is enriched at the AIS of cultured neurons prior to detergent extraction (Fig. 7g). Ranbp2, although traditionally thought to be part of the nuclear pore complex^47^, may participate in a trafficking complex, including Klc1 (Fig. S4e–h) and the kinesins Kif5b and Kif5c^48^. Similarly, Macf1 (*Macf1*; microtubule actin cross-linking factor 1) was preferentially biotinylated by Ndel1C-BirA*, but not NF186-BirA*. We determined Macf1’s location since it coordinates interactions between microtubules and F-actin, and regulates polarization of cells^49^. Immunolabeling of Macf1 revealed a detergent-resistant pool at the AIS in 92% of neurons examined (Fig. 7h). These results show that proximity biotinylation using AIS-targeted Ndel1C can reveal AIS proteins and map their relative locations in the AIS.

An important consideration with this experimental design, compared to that using NF186-BirA* and NF186∆FIGQY-BirA* chimeras, is that both Ndel1C chimeras localize to the AIS. Thus, proteins that are in equal proximity to the BirA* on Ndel1C N- and C-termini, i.e., close to the middle of Ndel1C, will not be preferentially biotinylated by one specific chimera and instead will lie along the least-squares fit line and within our 10 PSM cutoff range (Supplementary Fig. 5a, b). Many of these common proteins with the highest number of peptides were tubulins (Fig. 7c).

### Trim46 proximity proteome maps AIS microtubules

**Fig. 8:**
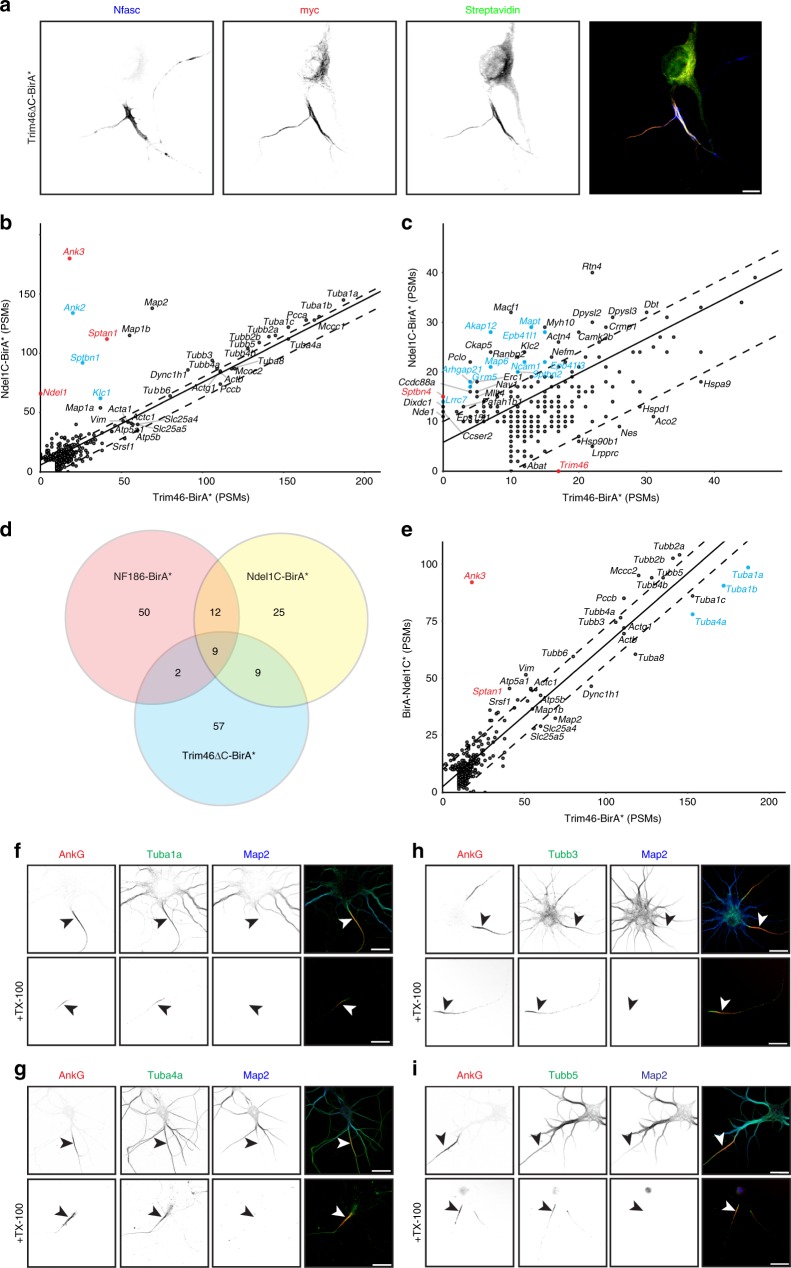
The Trim46 proximity proteome reveals AIS tubulins. **a** Immunolabeling of DIV14 hippocampal neurons transduced at DIV11 using adenovirus to express myc-tagged Trim46∆C-BirA*. From DIV13, neuronal culture media included 50 µM biotin. AIS are labeled using antibodies against Neurofascin (Nfasc, blue). The Trim46∆C-BirA* chimera is labeled using anti-myc antibodies (red). Biotinylated proteins were detected using Alexa488-conjugated Streptavidin. Scale bar = 5 µm. **b**, **c** Comparison of the peptide spectral matches (PSMs) for each protein biotinylated by Ndel1C-BirA* and proteins identified using combined Trim46∆C-BirA* and BirA*-Trim46∆C chimeras (referred to as Trim46-BirA*); panels **b** and **c** are shown at different scales. Known AIS proteins are indicated in red. Proteins enriched and previously identified as candidate AIS proteins in the NF186-BirA* samples are indicated in blue. The solid line is the least-squares fit of the biotinylated proteins while the dotted lines parallel to the least-squares fit line represent an arbitrary minimum of ten PSMs confidence interval. **d** Venn-diagram illustrating the number of candidate AIS proteins identified in each proximity proteome. **e** Comparison of the PSMs for each protein biotinylated by BirA*-Ndel1C and proteins identified using combined Trim46∆C-BirA* and BirA*-Trim46∆C chimeras. Known AIS proteins are indicated in red. Proteins enriched and previously identified as candidate AIS proteins in the NF186-BirA* samples are indicated in blue. The solid line is the least-squares fit of the biotinylated proteins while the dotted lines parallel to the least-squares fit line represent an arbitrary minimum of ten PSMs confidence interval. **f**–**i** Immunostaining of DIV14 cultured hippocampal neurons using antibodies against AnkG (red), Tuba1a (**f**, green), Tuba4a (**g**, green), Tubb3 (**h**, green), Tubb5 (**i**, green), and Map2 (blue). The lower panels show the Triton X-100 detergent-resistant pools of the indicated tubulins. AIS are indicated by arrowheads. Scalebars = 10 µm.

One unique structural feature of the AIS is the presence of fasciculated microtubules^50^; these microtubules are distributed through the AIS (average depth of ~85 nm^42^). Trim46 is enriched at the AIS, regulates neuronal polarity, and bundles microtubules (Fig. 7a); a truncated Trim46 lacking its C-terminus (Trim46∆C) is sufficient to localize at the AIS^19^. However, the amino acids mediating Trim46’s AIS localization are unknown. Therefore, to identify proteins that participate in AIS microtubule functions, AIS microtubule composition, and to probe the molecular organization of the fasciculated microtubule domain, we used the same strategy as we did for Ndel1: we constructed myc-tagged Trim46∆C-BirA* and BirA*-Trim46∆C chimeras. We packaged these in adenovirus, and transduced DIV11 neurons. By DIV14, we found Trim46∆C-BirA* and BirA*-Trim46∆C were enriched at the AIS, but did not precisely colocalize with NF186 since NF186 is a transmembrane protein while Trim46∆C chimeras decorate the AIS microtubule cytoskeleton (Fig. 8a, BirA*-Trim46∆C is not shown). Addition of biotin at DIV13 caused biotinylation of the AIS microtubule compartment by DIV14 (Fig. 8a). We transduced neurons using the Trim46∆C-BirA* or BirA*-Trim46∆C chimeras, and then performed mass spectrometry on biotinylated proteins. After removing the endogenously biotinylated carboxylases, we found that biotinylated proteins, most prominently tubulins, were found near the least-squares fit line (Supplementary Fig. 6a). Interestingly, when we identified proteins that were preferentially biotinylated by Trim46∆C-BirA* (twofold over BirA*-Trim46∆C; Supplementary Fig. 6b), we found that 2/77 are known AIS proteins (Fig. 8d and Supplementary 6b, red), 9/77 are also preferentially biotinylated by both NF186-BirA* and Ndel1C-BirA* (Fig. 8d and Supplementary Fig. 6b, blue), and 9/77 are also preferentially biotinylated by Ndel1C-BirA* (Fig. 8d and Supplementary Fig. 6b, green). Thus, proximity biotinylation using Trim46∆C-BirA* and BirA*-Trim46∆C further defines the molecular organization of the AIS.

To gain additional insight into the microtubule compartment of the AIS, we performed a separate experiment where we pooled proteins biotinylated by Trim46∆C-BirA* and BirA*- Trim46∆C (collectively referred to as Trim46-BirA*). We compared these biotinylated proteins to proteins biotinylated in sister cultures expressing BirA*-Ndel1C or Ndel1C-BirA* chimeras, since our results suggest the Ndel1C-BirA* is oriented toward AnkG, while BirA*-Ndel1C is oriented away from AnkG. Comparison of Ndel1C-BirA* with Trim46-BirA* showed Ndel1C-BirA* preferentially biotinylated known AIS proteins (red) and proteins we identified as interacting with AnkG (e.g., β4 spectrin and Map6; Fig. 8b, c).

Since Ndel1 regulates dynein activity along microtubules, and BirA*-Ndel1C is oriented with the BirA* away from AnkG (Fig. 7), we considered whether BirA*-Ndel1C and Trim46-BirA* chimeras biotinylate a similar pool of proteins, resulting in most identified proteins near the least-squares fit line (Fig. 8e). Similar to our results comparing Trim46∆C-BirA* and BirA*-Trim46∆C (Supplementary Fig. 6a), we found many tubulin subunits were biotinylated by both Trim46-BirA* and BirA*-Ndel1C.

Since many tubulin subunits were identified using the non-AIS-targeting chimera NF186∆FIGQY-BirA* (Fig. 1c) or BirA* alone (Supplementary Fig. 3a), it is possible that tubulins are simply non-specifically biotinylated proteins in the Trim46-BirA* experiments. However, tubulins are part of the AIS cytoskeleton. Therefore, to determine which tubulins are retained as part of the AIS cytoskeleton, we immunostained cultured hippocampal neurons with, and without prior detergent extraction, using tubulin antibodies. We found that α tubulins (Tuba1a (*Tuba1a*) and Tuba4a (*Tuba4a*)) and β-tubulins (Tubb3 (*Tubb3*) and Tubb5 (*Tubb5*)) labeled both axonal and somatodendritic compartments (Fig. 7f–i). However, 95%, 99%, 97%, and 98% of neurons also had a detergent-resistant AIS pool of Tuba1a, Tuba4a, Tubb3, and Tubb5, respectively (Fig. 7f–i, arrowheads). Thus, proximity biotinylation using Ndel1C and Trim46∆C chimeras reveal tubulin subunits that may be part of the unique microtubule cytoskeleton found at the AIS.

## Discussion

We used molecular targeting of the biotin-ligase BirA* to map the molecular organization of the AIS. BirA* can be used both in vitro and in vivo to identify proteomes based on proximity. For example, Kim et al.^23^ used BirA* in HEK293 cells to investigate the nuclear pore complex, while Uezu et al.^43^ targeted BirA* in vivo to excitatory and inhibitory synapses using PSD-95-BirA* and Gephyrin-BirA* chimeras, respectively. The experiments in neurons yielded robust proteomes for inhibitory and excitatory synapses. Other methods for proximity biotinylation (e.g., APEX) also provided important insights into synapse structure^51^. However, proximity biotinylation using BirA* or APEX is limited to a range of ~10 nm. Thus, for structures larger than a post-synaptic density (e.g., AIS), a multiplexed approach is necessary to improve coverage and specificity. For example, we found significant overlap between the proteins biotinylated by NF186-BirA* and Ndel1C-BirA* (Fig. 8d). Discovery of the same proteins using independent AIS-targeting strategies significantly increases our confidence that the identified proteins are AIS proteins.

This multiplexed approach can be replicated in other subcellular compartments and domains (e.g., organelles, growth cones, etc.), and our experiments in neurons serve as a paradigm. Our experiments at the AIS also emphasize the importance of appropriate controls and experimental design. Here, we used two separate approaches: a non-targeting chimera, and chimeras with BirA* attached to different locations of the bait protein. We found both methods effectively revealed AIS proteins, but also missed several known AIS membrane and membrane-associated proteins, including Nav1.6, NrCAM, KCNQ2/3K^+^ channels, Caspr2, Kv1 K^+^ channels, and PSD-93^12,52^. The limited range of BirA* may account for the absence of some of these AIS proteins, and reinforces the importance of using multiple independent chimeras to improve coverage of the AIS protein landscape. In some instances, membrane proteins were missed due to our stringent peptide cutoff. Application of extracellular proximity biotinylation might also yield better representation of membrane and membrane-associated proteins, including AIS-enriched extracellular matrix molecules^53^. Alternatively, we may have missed some AIS membrane proteins since we collected biotinylated proteins at DIV14, before most AIS are fully mature. Thus, future experiments using NF186-BirA* and NF186∆FIGQY-BirA* chimeras at different developmental stages, together with AIS-specific extracellular proximity biotinylation, may uncover AIS proteins that are developmentally regulated or that play key roles in later events, including the formation of AIS axo-axonic synapses^54^. The selective purification of spatially distinct pools of proteins using proximity biotinylation and mass spectrometry can also be used to examine post-translational modifications. For example, the AIS is a hotspot for protein phosphorylation^18,55,56^, and subsequent analysis of biotinylated AIS proteins may define the specific phosphorylation sites, among other modifications.

The AIS is highly enriched with cytoskeletal proteins, including Ankyrins, Spectrins, actin, and tubulins. Since the chimeras we used all relied on interaction with this cytoskeleton, it is not surprising that the AIS proteins we identified are related to cytoskeletal functions. The close association with AIS cytoskeletal proteins renders many AIS proteins detergent insoluble, and we used this as one characteristic of AIS proteins. An important advantage of our AIS-restricted proximity biotinylation strategy is that it allowed us to identify proteins that participate in AIS structure and function, but that are not found exclusively at the AIS. This vastly expands the repertoire of proteins that can be investigated to determine the mechanisms regulating AIS functions, including neuronal polarity and trafficking of axonal and dendritic vesicles and organelles.

Our experiments provide an important resource for future studies of AIS structure and function. Many of the identified proteins suggest mechanisms regulating protein trafficking, AIS stability, and cytoskeletal plasticity. For example, we showed that one of the most highly biotinylated proteins using the NF186-BirA* chimera is Mical3. Mical3 interacts with AIS spectrins, a detergent-resistant subset of Mical3 is located at the AIS, and knockdown of Mical3 resulted in accumulation of actin at the AIS. Further, our experiments suggest that activation of Mical3 by Rab proteins alters the structure of AIS actin patches. Thus, we speculate that Mical3 may help regulate the differential trafficking of proteins and organelles in neurons^17^. Future studies will be needed to verify and better define this mechanism. It is likely that a Mical3-dependent mechanism is but one of many involved in sorting of cargoes in the AIS. For example, the identification of Ranbp2 and AnkG-interacting Klc1 at the AIS, both of which have been reported to participate in a trafficking complex in other cells^48^, points to yet a different mechanism that deserves additional investigation.

The discovery of AIS Septins is consistent with their recognized roles in cytoskeletal remodeling and polarity^41^. Septin filaments can form membrane boundaries that restrict the lateral diffusion of membrane proteins and lipids^57^. Since we mainly identified Septins using the NF186-BirA* chimera, this suggests they are in a privileged location and not accessible to biotinylation by Ndel1C-BirA*. We previously reported that AnkG is restricted to the AIS through the formation of an intra-axonal membrane boundary made by AnkB and β2 spectrin. Since NF186-BirA* biotinylated both AnkB and β2 spectrin, Septins may also function in the distal axon. One previous study of Septins in neurons focused on Septins 3, 5, 6, 7, and 11 (the same Septins we found in our experiments) since their expression increases during development and they are highly expressed in axons. Consistent with an important role in axons, reducing expression of Sept5 or Sept7 by shRNA disrupted axon growth^58^. Here, we showed that loss of Septins disrupted the AnkG-dependent AIS cytoskeleton. However, in mature neurons the AIS microtubule compartment defined by Trim46 was initially resistant to disruption, suggesting that AnkG’s remarkable stability and low turnover rate^59^ may reflect its interactions with Septins at the plasma membrane.

Mical3, Septins, Map6, Klc1, Ranbp2, and Macf1 are but a few of the AIS proteins identified and verified in our experiments. Their discovery at the AIS demonstrates that targeted and multiplexed proximity biotinylation is a powerful approach to spatially and molecularly map subcellular proteomes. The AIS proteome reported here also includes many other interesting proteins with functions that may be important at the AIS, but that we have not investigated. For example, members of the collapsin response mediator protein (Crmp) family were preferentially biotinylated by NF186-BirA* (Crmp2 (*Dpysl2*), Crmp3 (*Dpysl4*) and Crmp5 (*Dpysl5*)), Ndel1C-BirA* (Crmp3 (*Dpysl4*) and Crmp5 (*Dpysl5*)), and Trim46∆C-BirA* (Crmp2(*Dpysl2*) and Crmp4 (*Dpysl3*)). Crmp2 is a key regulator of neuronal polarity^60^, and a recent report showed that loss of AnkG promotes microtubule instability through phosphorylation of CRMP2^61^. Crmp2 also binds directly to Klc1^62^. Thus, members of the Crmp family may play many, as yet undiscovered, roles at the AIS.

The NF186, Ndel1, and Trim46 proximity proteomes led to conceptual insights for mechanisms regulating AIS structure and function. We suggest our results should be used as a path forward by neurobiologists interested in the molecular mechanisms whereby AIS regulate protein trafficking, neuronal polarity, and axon function.

## Methods

### Animals

Timed pregnant Sprague-Dawley rats were obtained from Harlan Sprague-Dawley. Rats were killed for embryo collection at E18. All experiments were performed in compliance with the National Institutes of Health Guide for the Care and Use of Laboratory Animals and were approved by the Baylor College of Medicine Institutional Animal Care and Use Committee.

### Adenovirus production

ViraPower Adenoviral Promoterless Gateway Expression Kit (Invitrogen) was used to construct adenovirus. The pENTR11 shuttle vector was modified by adding the human neuron-specific enolase promoter (hENO2) from Addgene (Plasmid #11606). The hENO2 promoter was amplified by PCR and inserted into pENTR11 and named pENTR11-hENO2-MCS. BirA* was amplified by PCR from pcDNA3.1 Myc-BirA(R118G)-MCS (Addgene, Plasmid #35700) to generate a shuttle template named pENTR11-hENO2-MCS-BirA*-MCS. To generate pENTR11-hENO2-HA-NF186-Myc-BirA* and pENTR11-hENO2-HA-NF186∆FIGQY-Myc-BirA*, complementary DNA (cDNA) encoding HA-NF186 and NF186∆FIGQY were amplified by PCR from Rat NF186 tagged with an N-terminal HA epitope tag^53^. pENTR11-hENO2-Ndel1C-Myc-BirA* and pENTR11-hENO2-Myc-BirA*-Ndel1C were generated by amplifying Ndel1C (Origene) and inserting it into shuttle template pENTR11-hENO2-MCS-BirA*-MCS. pENTR11-hENO2-Trim46∆C -Myc-BirA* and pENTR11-hENO2-Myc-BirA*- Trim46∆C were generated by amplifying Trim46 (Origene) and inserting it into shuttle template pENTR11-hENO2-MCS-BirA*-MCS. All shuttle vectors were cloned into pAd/PL-DEST (Gateway destination vector) by LR recombination reaction between each entry clone (pENTR11-hEno2-HA-NF186-Myc-BirA*, pENTR11-hENO2-HA-NF186ΔFIGQY-Myc-BirA*, pENTR11-hENO2-Ndel1C-Myc-BirA*, pENTR11-hENO2-Myc-BirA*-Ndel1C, pENTR11-hENO2-Trim46∆C-Myc-BirA*, and pENTR11-hENO2-Myc-BirA*-Trim46∆C) and a pAd/PL-DEST according to the manufacturer’s instruction (Life Technologies) to generate pAd/PL-DEST-hEno2-HA-NF186-Myc-BirA*, pAd/PL-DEST-hEno2-HA-NF186ΔFIGQY -Myc-BirA*, pAd/PL-DEST-hENO2-Ndel1C-Myc-BirA*, pAd/PL-DEST-hENO2-Myc-BirA*-Ndel1C, pAd/PL-DEST-hENO2-Trim46∆C-Myc-BirA*, and pAd/PL-DEST-hENO2-Myc-BirA*-Trim46∆C, respectively. The expression clones were digested with Pac I to expose the viral inverted terminal repeats (ITRs). First generation adenoviruses were produced as described in ref. ^63^. Briefly, adenoviral vectors were rescued by digesting the adenoviral plasmids with PacI followed by transfection into 293 cells^64^ by calcium phosphate co-precipitation. Individual, well isolated plaques were picked and expanded by infecting 293 cells. 293N3S cells^65^ were used for large scale adenoviral vector production in spinner cultures.

### Plasmids

Plasmids for mouse Sept3, Sept5, Sept6, Sept7, Sept11, KLC1, and MsrB1 were purchased from Origene. GFP-UtrCH plasmid was from Addgene. α2-spectrin-GFP was gift from Dr. Michael Stankewich (Yale University). Mouse Mical3-6xMyc, was a gift from Dr. Alex Kolodkin (Johns Hopkins University). Macf1-GFP was a gift from Dr. Elaine Fuchs (Rockefeller University). The full-length β4 Σ1-spectrin with N-terminal myc tag was subcloned and inserted into pCS3 + MT expression vector. Rab8a, Rab8b, Rab13, Rab35, Rab36 cDNAs were amplified by PCR using a mouse brain cDNA library and inserted into pTagRFPC1 vector (Evrogen).

### shRNA plasmid construction

The 19 or 21-nucleotide shRNA oligos were synthesized by Millipore Sigma and annealed. The shRNAs were inserted into the *Bgl*II and *Hin*dIII sites of GFP-expressing pSuper vector. The specific target sequence of shRNA for *P. pyralis* luciferase was: 5ʹ-CGCTGAGTACTTCGAAATGTC-3ʹ; Septin3 was targeted with Sept3-shRNA#1 (5ʹ-GACAGTGGAGATCAAAGCGAT-3ʹ); Septin5 was targeted with Sept5-shRNA#1 (5ʹ-GCGGTGAACAACTCTGAATGT-3ʹ); Septin6 was targeted with Sept6-shRNA#1 (5ʹ-GCCCATCGTGGAATTCATTGA-3ʹ); Septin7 was targeted with Sept7-shRNA#1 (5ʹ-GATAACAGGGTGCAGTGTTGT-3ʹ); Septin11 was targeted with Sept11-shRNA#1 (5ʹ-GGGATTTGGAGACCAGATTAA-3ʹ); Map6 was targeted with Map6-shRNA#1 (5ʹ-GGTGCAGATCAGCGTGACA-3ʹ); Map6-shRNA#2 (5ʹ-GCTCCTACAGGAACGAATTCA-3ʹ); Klc1 was targeted with Klc1-shRNA (5ʹ-GGTGATGATGGCCCTGTCTAA-3ʹ); Mical3 was targeted with Mical3-shRNA#1 (5ʹ-GCAGAAAGGGAAAGCATTTAT-3ʹ), and Mical3-shRNA#2 (5ʹ-CCGCAGGAATAAGAAAGAGAAGAAA-3ʹ). Plasmid DNA and shRNA constructs were verified by sequencing (Genewiz or GenScript). Primers for construction of plasmids are included in the Supplementary Methods.

### Antibodies

The following antibodies were used (dilutions for each antibody are indicated in parentheses): mouse monoclonal antibody anti-Septin 3 (1:200; Millipore Sigma Cat# MABN1153), rabbit polyclonal antibody anti-Septin 5 (1:200; IBL-America Cat# 018921, RRID:AB_1540782), rabbit polyclonal antibody anti-Septin 6 (1:500; Santa Cruz Biotechnology Cat# sc-20180, RRID:AB_2184999), rabbit polyclonal antibody anti-Septin 7 (1:200; IBL-America Cat# JP18991, RRID:AB_1630825), rabbit polyclonal antibody anti-Septin 11 (1:200; Millipore Sigma Cat# ABN1342), mouse monoclonal antibody anti-FLAG (1:500; Millipore Sigma Cat# F1804, RRIB:AB_262044), mouse monoclonal antibody anti-c-Myc (1:1000; BioLegend, Cat# 626802, RRID:AB_2148451), mouse monoclonal antibody anti-HA (1:1000; BioLegend Cat# 901503, RRID:AB_2565005), rabbit polycolonal antibody anti-β4-spectrin (1:500; RRID:AB_2315634) was generated against the peptide sequence DRAEELPRRRRPERQE (in the C-terminal “specific domain” of β4 spectrin), mouse monoclonal antibody anti-β2-spectrin (1:1000; B&D Systems Cat# 612563, RRID:AB_399854), mouse monoclonal antibody anti-α2-spectrin (1:1000; BioLegend Cat# 803201, RRID:AB_2564660). Mouse monoclonal anti-AnkyrinG (1:500; Cat# N106/36, RRID:AB_10673030 and N106/65, RRID:AB_10675130), anti-Neurofascin (1:500; A12/18, AB_2282826), and anti-GST (1:2000; N100/13, AB_10671818) antibodies were purchased from the UC Davis/NIH NeuroMab Facility (Davis, CA). Rabbit polyclonal antibody anti-GFP (1:700; Thermo Fisher Scientific, Cat# A-11122, RRID:AB_221569), mouse monoclonal antibody anti-Klc1 (1:500; Santa Cruz Biotechnology Cat# sc-25735, RRID:AB_2280879), mouse monoclonal antibody anti-Kif5A (1:1000; Santa Cruz Biotechnology Cat# sc-376452, RRID:AB_11150294), rabbit polyclonal antibody anti-Macf1 (1:200; Santa Cruz Biotechnology Cat# sc-68428, RRID:AB_2139238), rabbit polyclonal antibody anti-Ranbp2 (1:200; Bethyl Cat# A301-796A, RRID:AB_1211503), rabbit anti-Mical3 (1:200; a gift from Dr. Anna Akhmanova, Utrecht University), mouse monoclonal antibody anti-Mical3 (1:500; a gift from Dr. Alex Kolodkin, Johns Hopkins University), mouse monoclonal antibody anti-Map6 (1:400; Cell Signaling Technology Cat# 4265, RRID:AB_2140993), mouse monoclonal antibody anti-Tuba1a (1:1000; Millipore Sigma Cat# SAB1411824), rabbit monoclonal antibody anti-Tuba4a (1:1000; abcam Cat# EPR13477(B)), mouse monoclonal antibody to Tubb3 (1:1000; Millipore Cat# MAB1637, RRID:AB_2210524), mouse monoclonal antibody anti-Tubb5 (1:1000; Millipore Sigma, Cat# SAB1305556), rabbit polyclonal antibody anti-GAPDH (1:2000; Millipore Sigma Cat#G9545, RRID:AB_796208). Chicken antibodies to: MAP2 (1:1000; EnCor, Cat#CACP-MAP2, RRID:AB_2138173) and Neurofascin (1:1000; R&D Systems Cat#AF3235, RRID:AB_10890736). Aminomethylcoumarin (AMCA), Alexa Fluor 488, and Alexa Fluor 594 conjugated secondary antibodies were purchased from Thermo Fisher Scientific (all 1:1000). Anti-mouse horseradish peroxidase (HRP)-labeled secondary antibody (1:1000; GE Healthcare, Cat# NA9310V, RRID:AB_7721983) and anti-rabbit HRP antibody (1:1000; 111-035-003, Jackson Laboratory; RRID:AB_2313567). Streptavidin, Alexa Fluor 488 and 594 conjugates were purchased from Thermo Fisher Scientific (1:2000; Cat# S11223, RRID:AB_2336881 and S11227, RRID:AB_2313574).

### Visualization of actin patches

For actin visualization, DIV 14 cells were fixed with 4% paraformaldehyde pH = 7.5 for 10 min, then permeabilized for 1 h PBGTS 0.1% Triton, followed by 1 h of dye-loading with 100 nM Acti-stain 488 Fluorescent Phalloidin (Amanita phalloides) (Cytoskeleton, Inc, Denver, CO Cat# PHDG1), Acti-stain 555 Fluorescent Phalloidin (Amanita phalloides) Cytoskeleton, Inc, Denver, CO Cat# PHDH1), taken from a 14 μM stock prepared according to the manufacturer’s instructions. Staining of neurons was done on all cultures at the same time and with the same reagents for each experiment. To count actin patches, experimenters were blinded to the plasmid transfection (except for the case of no transfection since there was no red fluorescent protein (RFP) signal). Images of AIS in neurons with RFP were then taken at 63×. The images were then randomized, and the number of actin patches along each AIS were counted. Each punctum of phalloidin fluorescence was counted as an actin patch.

### Cell culture

Primary cultures of hippocampal neurons were obtained from E18 Sprague-Dawley rat embryos. Hippocampi were dissected and dissociated. Neurons were then plated onto Poly-d-Lysine (Sigma) and laminin-coated glass coverslips (Life Technologies) at a density of 4800 cells/cm^2^. Hippocampal neurons were cultured in Neurobasal medium (Life Technologies) containing 1% Glutamax (Life Technologies), 1% penicillin and streptomycin (Life Technologies), and 2% B27 supplement (Life Technologies) in an incubator with 5% CO_2_. Transfection of hippocampal neurons was performed using Lipofectamine 2000^53^. Human embryonic kidney (HEK293T) (Cat#CRL-3216, RRID:CVCL_0063) cells and COS7 Cells (Cat#CRL-1651, RRID:CVCL_0224) were purchased from ATCC and cultured on cell culture dishes at 37 °C in Dulbecco’s Modified Eagle Media (DMEM) containing 10% heat-inactivated Fetal bovine serum (GE Healthcare), 50 units/ml penicillin, and 50 μg/ml streptomycin.

### Plasmid transfection

HEK293T cells and COS7 cells were transfected with plasmid DNA using Lipofectamine 3000 (Invitrogen) according to the manufacturer’s instructions. The media was replaced after 24 h of transfection. Primary hippocampal neurons were transfected with Lipofectamine 2000 (Invitrogen). After neurons were washed with neurobasal media three times, the media was replaced after 5 h of transfection.

### Immunoblotting and Immunoprecipitation

HEK293T cells were transfected with plasmid DNA using the Lipofectamine 3000 transfection reagent. After 48 h of transfection, cells were lysed in RIPA buffer (50 mM Tris-HCl, 150 mM NaCl, 0.5% sodium deoxycholate, 0.1% SDS, and 1% NP-40) or lysis buffer (50 mM Tris-HCl (pH8.0), 150 mM NaCl, 1 mM EDTA, 0.1% Triton X-100) and the lysates were centrifuged at 13,000 × *g* for 10 min at 4 °C. Alternatively, brain homogenate was lysed in RIPA buffer. Lysates were mixed with protein G resin (GE Healthcare) preabsorbed with each of the primary antibodies. The immune complexes were precipitated by centrifugation and washed three times with RIPA or lysis buffer. The immunoprecipitants were boiled in sample buffer and then separated by sodium dodecyl sulfate–polyacrylamide gel electrophoresis (SDS-PAGE). The proteins in supernatants were denatured in Laemmli sample buffer and then subjected to SDS-PAGE. The electrophoretically separated proteins were transferred to a polyvinylidene difluoride membrane, blocked with 5% skim milk, and immunoblotted with each of the primary antibodies and in turn with peroxidase-conjugated secondary antibodies. Original immunoblots used here are included in the source data file.

For α2 spectrin GST fusion protein pulldowns, HEK293T cells were transfected with plasmid DNA (Mical3-myc + α2 spectrin GST fusion protein) using the Lipofectamine 3000 transfection reagent. After 24 h of transfection, cells were lysed in lysis buffer (150 mM NaCl, 20 mM Tris-HCl (ph8.0), 10 mM EDTA, and 1% Triton X-100) and the lysates were centrifuged at 13,000 × *g* for 10 min at 4 °C. Lysates were mixed with glutathione sepharose 4B beads (GE Healthcare) preblocked in 10 mg/ml BSA overnight at 4 °C. Beads and depleted supernatants were boiled in reducing sample buffer and proteins were separated by SDS-PAGE. The electrophoretically separated proteins were transferred to nitrocellulose membranes and blocked with 5% skim milk. Membranes were immunoblotted with mouse anti-myc or mouse anti-GST primary antibodies, followed by HRP-conjugated goat anti-mouse secondary antibodies.

### In vitro biotinylation

Cultured rat primary neurons (≅ 20 × 10^6^ primary hippocampal neurons for each virus used) were transduced using the following adenoviruses; Ad-hENO2-HA-NF186-Myc-BirA*, Ad-hENO2-HA-NF186∆FIGQY-Myc-BirA*, Ad-hENO2-Ndel1C-Myc-BirA*, Ad-hENO2-Myc-BirA*-Ndel1C, Ad-hENO2-Trim46∆C -Myc-BirA*, and Ad-hENO2-Myc-BirA*-Trim46∆C at DIV11 with titer concentration 7 MOI for each virus. Biotin was added at DIV13 at a final concentration of 50 μM (Millipore Sigma Cat# B4639). Cells were collected at DIV14 and were lysed in RIPA buffer (50 mM Tris-HCl, 150 mM NaCl, 0.5% sodium deoxycholate, 0.1% SDS, and 1% NP-40). Biotinylated proteins were isolated using Streptavidin magnetic sepharose beads (GE Healthcare) overnight at 4 °C and then beads washed three times in 50 mM Tris buffered saline pH = 7.5.

### Mass spectrometry

Sample-incubated streptavidin magnetic sepharose beads were resuspended in 5 mM DTT in 100 mM NH_4_HCO_3_ and incubated for 30 min at room temperature. After this, iodoacetamide was added to a final concentration of 7.5 mM and samples incubated for 30 additional minutes. In all, 0.5 μg of sequencing grade trypsin (Promega) was added to each sample and incubated at 37 °C overnight. Supernatants of the beads were recovered, and beads digested again using 0.5 μg trypsin in 100 mM NH_4_HCO_3_ for 2 h. Peptides from both consecutive digestions were recovered by solid phase extraction using C18 ZipTips (Millipore), and resuspended in 0.1% formic acid for analysis by liquid chromatography–mass spectrometry (LC-MS/MS). Peptides resulting from trypsinization were analyzed on a QExactive Plus (Thermo Scientific), connected to a NanoAcquity™ Ultra Performance UPLC system (Waters). A 15-cm EasySpray C18 column (Thermo Scientific) was used to resolve peptides (90-min gradient with 0.1% formic acid in water as mobile phase A and 0.1% formic acid in acetonitrile as mobile phase B). MS was operated in data-dependent mode to automatically switch between MS and MS/MS. The top ten precursor ions with a charge state of 2+ or higher were fragmented by high-energy collisional dissociation. Peak lists were generated using PAVA software^66^. All generated peak lists were searched against the rat subset of the UniProt database (UniprotKB 2017.11.01) using Protein Prospector^67^. The database search was performed with the following parameters: a mass tolerance of 20 ppm for precursor masses; 30 ppm for MS/MS, cysteine carbamidomethylation as a fixed modification and acetylation of the N terminus of the protein, pyroglutamate formation from N-terminal glutamine, and oxidation of methionine as variable modifications. All spectra identified as matches to peptides of a given protein were reported, and the number of spectra (Peptide Spectral Matches, PSMs) used for label free quantitation of protein abundance in the samples.

### Protein networks analysis

The networks was constructed in Cytoscape using the STRING protein interaction database for mus musculus. Proteins were included if their fold change was >2 and at least ten peptides were found in one dataset. Interactions with a confidence score above 0.40 were included. Black line thickness was mapped to STRING interaction score and node size was set to the logarithm of the fold change. The size of the “Nfasc” node and node colors were manually set for esthetic purposes.

### Immunofluorescence microscopy

Cultured rat primary neurons were fixed in 4% paraformaldehyde (PFA, pH7.2) and immunostained with the indicated antibodies. In some instances, neurons were “extracted” prior to fixation using 0.5% TX-100 for 5 min at room temperature, or for 15 min at 4 °C. Images of immunofluorescence were captured using an Axio-imager Z1 microscope (Carl Zeiss MicroImaging) or Axio-observer Z1 microscope (Carl Zeiss MicroImaging) fitted with an AxioCam digital camera and Apotome for structured illumination (Carl Zeiss MicroImaging). Images were taken and collected by Zen (Carl Zeiss MicroImaging) acquisition software.

### Immunofluorescence analysis of AIS

After immunolabeling, we grouped shRNA-treated neurons according to whether they had an AIS, no AIS, or a disrupted AIS. “No AIS” included only neurons lacking AnkG in any processes. In some instances, AIS appeared fragmented or had dramatically reduced amounts of AIS AnkG compared to untransfected control neurons in the same dish. We counted these, pooled these with neurons lacking AIS, and considered them “no/disrupted AIS”. Experimenters were blinded to the experimental condition. For detergent extraction experiments, we subjectively determined if immunofluorescence for a candidate was above background and colocalized with AnkG.

### Single-molecule localization microscopy

Fourteen days in vitro (DIV) neurons were fixed using 4% PFA in PEM (80 mM Pipes, 5 mM EGTA, and 2 mM MgCl_2_, pH 6.8) for 10 min. After blocking, they were incubated with primary antibodies overnight at 4 °C, then with DNA-PAINT secondary antibodies (Ultivue) for 1 h at room temperature. DNA-PAINT imaging was performed on an N-STORM microscope (Nikon Instruments). The two channels were imaged sequentially in imaging buffer (PBS, 500 mM NaCl, 5% dextran sulfate) using the 647 nm laser at 40–60% power for 40,000 frames at 20 Hz. The first channel imaged was Mical3 with 0.26 nM imager I2-650, then α2-spectrin with 0.125 nM imager I1-650 (imagers from Ultivue).

### Statistics, reproducibility, and data presentation

All statistics were performed using Excel or GraphPad Prism software. Statistical tests are stated in each figure legend. Plots of mass spectrometry results were generated using R programming language. For all experiments involving immunofluorescence imaging, at least three independent experiments were performed with similar results; representative images are shown. The only exception to this was the SMLM of Mical3 where one independent experiment was performed. Nevertheless, many cells in this experiment were imaged with similar results. For immunoblots, the number of times each experiment was repeated varied from 1 to 3. For the blots shown in Figs. 3f and 4g, *n* = 1; for the blots shown in Fig. 5c and S4f, *n* = 2 experiments with similar results; for the blots shown in Figs. 3d, e and S4b, *n* = 3 experiments with similar results.

## Supplementary information

Supplementary Information

Description of Additional Supplementary Files

Supplementary Data 1

## Data Availability

All data generated or analyzed during this study, including mass spectrometry data sets, are available from the corresponding author upon request. All proteomics data have been deposited to the ProteomeXchange Consortium via the PRIDE partner repository with the dataset identifier PXD016272. A supplemental file including all PSMs is provided in Supplementary Data 1. The source data underlying Figs. 3d–f, 4b, d, g, 5g, 6b, d, i, j, and S4b, d, f, h are provided in the Source Data file.

## Source data

Source Data
