# Supplementary material for: Mapping axon initial segment structure and function by multiplexed proximity biotinylation
Source: Nat Commun. 2020 Jan 3;11:100. doi: 10.1038/s41467-019-13658-5 (PMC6941957; doi:10.1038/s41467-019-13658-5)
Supplement: Supplementary file 1 — Supplementary Information [file 41467_2019_13658_MOESM1_ESM.pdf]

Supplementary Information:

Mapping Axon Initial Segment Structure and Function by Multiplexed Proximity Biotinylation by Hamdan et al.

Supplementary information includes:

Supplementary Figures 1-6

Supplementary Methods

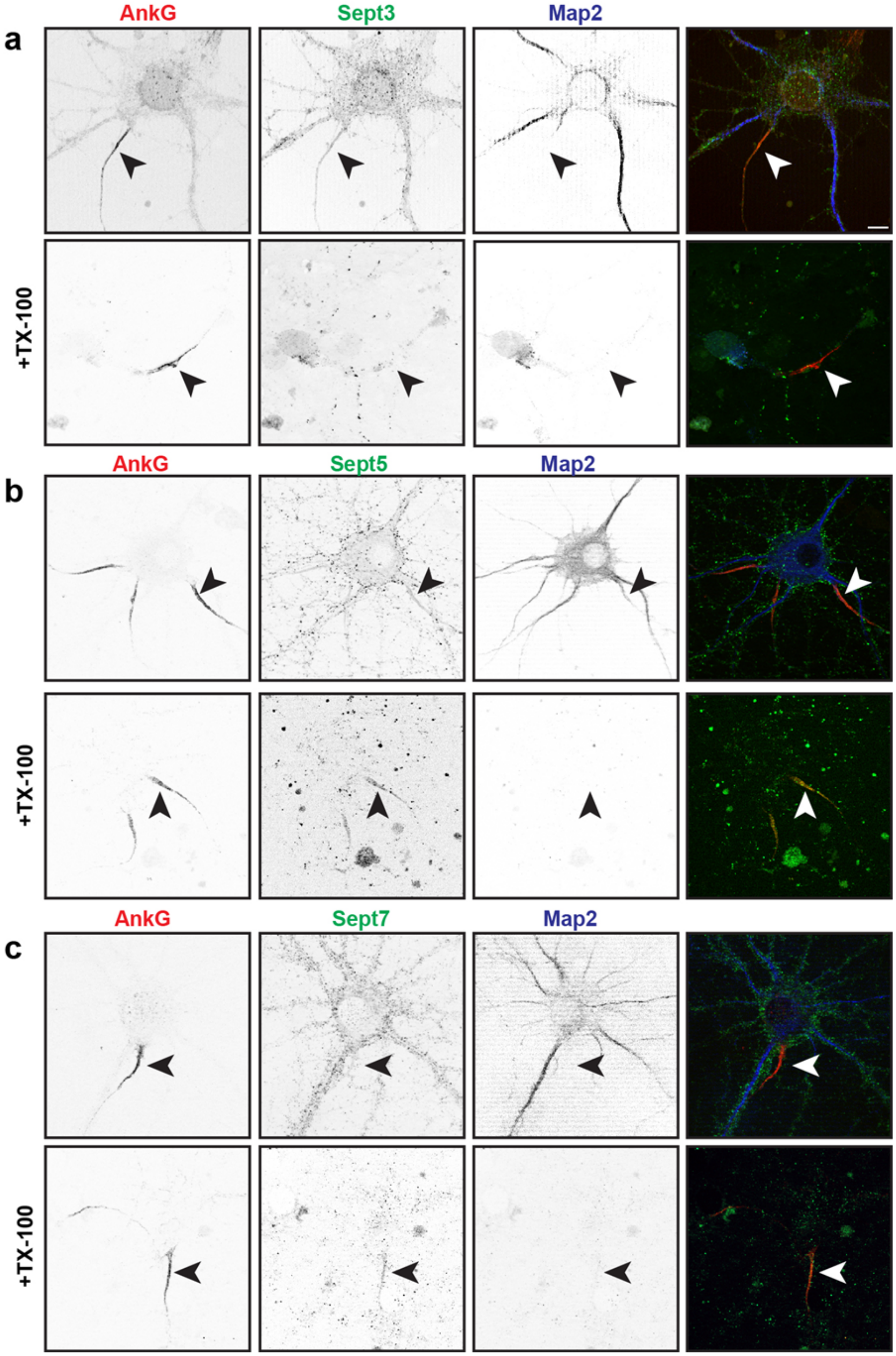

Supplementary Figure 1. A detergent insoluble pool of Septins is located at the AIS. (a-c) Immunostaining of DIV14 hippocampal neurons using antibodies against AnkG (red), Sept3 (a, green), Sept5 (b, green), Sept7 (c, green) and Map2 (blue). The lower panels show the Triton X-

100 detergent resistant pools of Sept5 (b) and Sept7 (c) at the AIS. AIS are indicated by arrowheads. Scale bar = 5 μm.

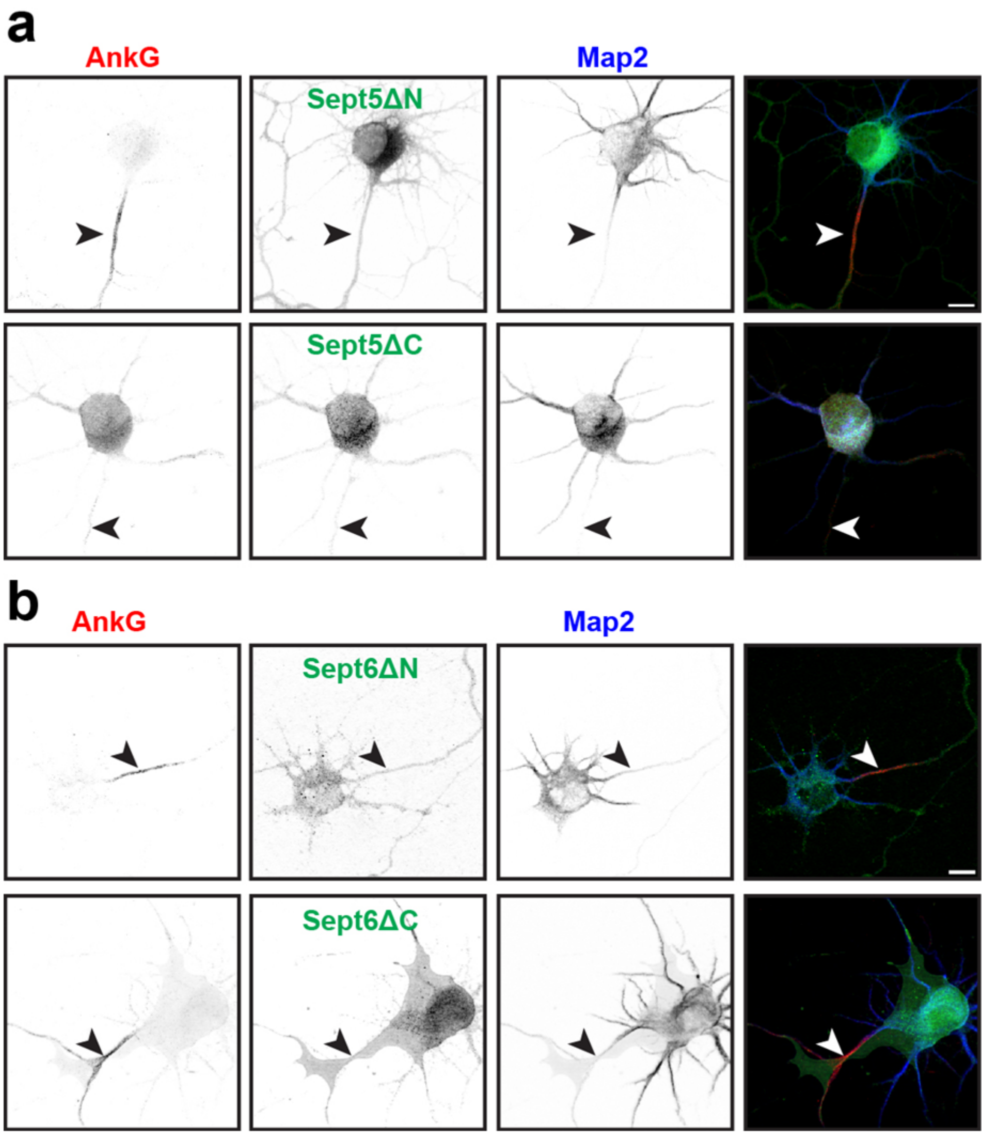

Supplementary Figure 2. The N-terminus of Sept5 binds to AnkG. (a) Immunostaining of neurons transfected using plasmids for Sept5ΔN (top) or Sept5ΔC (bottom) using antibodies against AnkG (red), Myc-tag (green), and Map2 (blue). AIS are indicated by arrowheads. Scalebar = 5 µm. (b) Immunostaining of neurons transfected using plasmids for Sept6ΔN (top) or Sept6ΔC (bottom) using antibodies against AnkG (red), Myc-tag (green), and Map2 (blue). AIS are indicated by arrowheads. Scalebar = 5 µm.

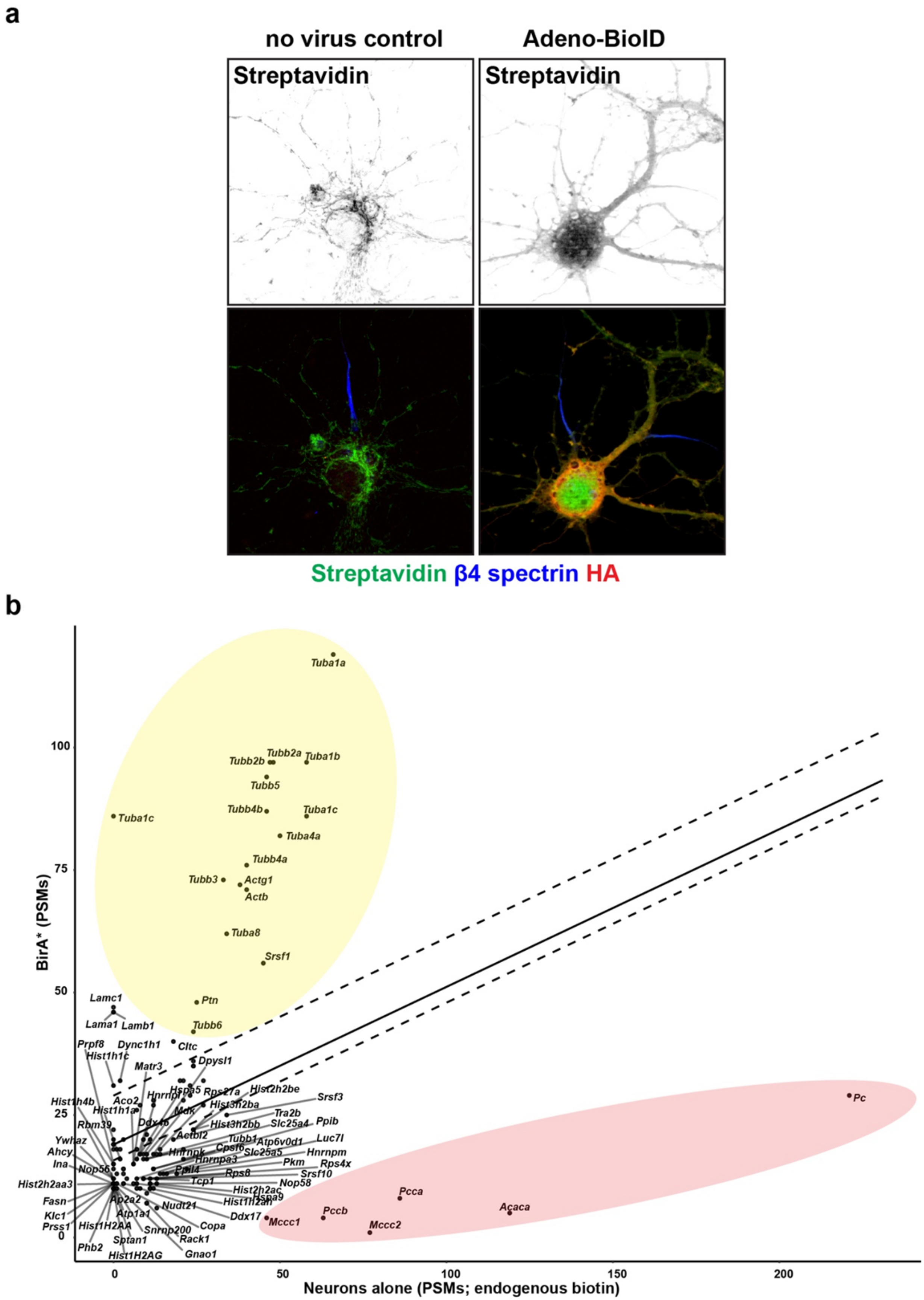

Supplementary Figure 3. Comparison of endogenously biotinylated proteins to those

biotinylated by BirA*-expressing neurons. (a) DIV 14 hippocampal neurons treated with 50 μM biotin for 24 hrs labeled using streptavidin (green) or antibodies against β4 spectrin (blue) or HA (red). HA detects the BirA* ligase in neurons transduced using an adenovirus to express HA-BioID. Scalebar = 10 μm. (b) The number of peptide spectral matches (PSMs) for each biotinylated protein identified by mass spectrometry. The solid line is the least-squares fit of the biotinylated proteins while the dotted lines parallel to the least-squares fit line represent an arbitrary minimum of 10 PSMs confidence interval. Endogenously biotinylated carboxylases are highlighted in the red region of the graph, while tubulins and actin are highly biotinyalted in BirA*-expressing neurons. For clarity, not all gene names are shown on the graph. N= 1 for BirA* transfected neurons and for untreated neurons.

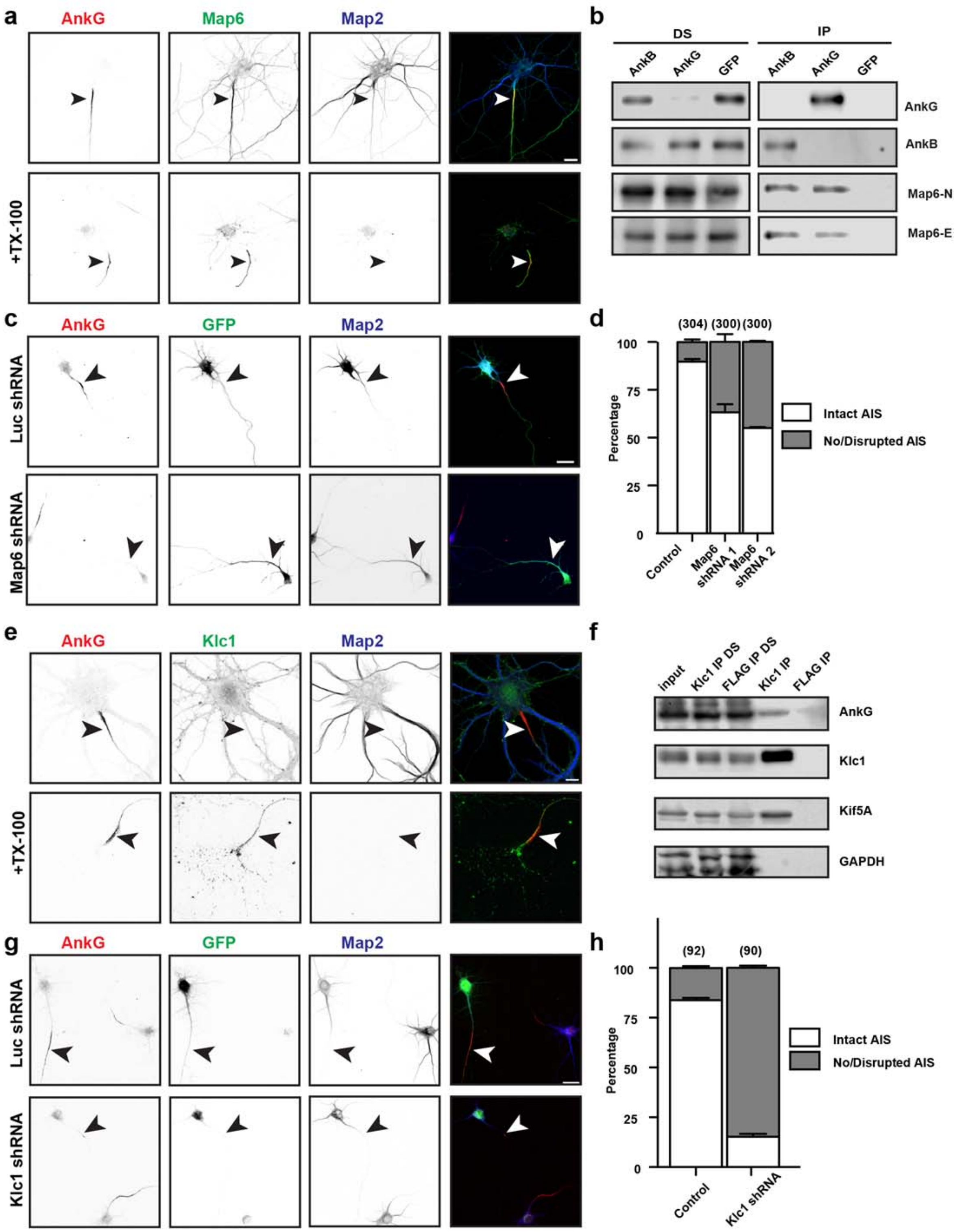

Supplementary Figure 4. Map6 and Klc1 are AIS proteins. (a) Immunostaining of DIV14 cultured hippocampal neurons using antibodies against AnkG (red), Map6 (green), and Map2

(blue). The lower panels show the Triton X-100 detergent resistant pool of Map6 at the AIS. AIS are indicated by arrowheads. Scalebar = 10 µm. (b) AnkG and AnkB can co-immunoprecipitate Map6 from brain homogenate. IP = immunoprecipitation, DS= depleted supernatant. (c) Immunostaining of DIV6 hippocampal neurons, transfected at DIV2 with control (Luciferase (Luc)) and Map6 shRNAs, using antibodies against AnkG (red), GFP (green), and Map2 (blue). AIS and proximal axons are indicated by arrowheads. Scalebar = 20 µm. (d) Quantification of the percentage of neurons with normal or no/disrupted AIS as indicated by AnkG immunostaining. Error bars, $\pm$SEM. N=3 independent experiments; the number of neurons counted is shown. (e) Immunostaining of DIV14 cultured hippocampal neurons using antibodies against AnkG (red), Klc1 (green), and Map2 (blue). The lower panels show the Triton X-100 detergent resistant pool of Klc1 at the AIS. AIS are indicated by arrowheads. Scalebar = 10 µm. (f) Klc1 can co-immunoprecipitate AnkG and Kif5A from brain homogenate. FLAG antibodies were used as a control. IP = immunoprecipitation, DS= depleted supernatant. (g) Immunostaining of DIV6 hippocampal neurons, transfected at DIV2 with control Luc shRNA and Klc1 shRNAs, using antibodies against AnkG (red), GFP (green), and Map2 (blue). AIS and proximal axons are indicated by arrowheads. Scalebar = 20 µm. (h) Quantification of the percentage of neurons with normal, disrupted, or no AIS as indicated by AnkG immunostaining. Error bars, $\pm$SEM. N=3 independent experiments; the number of neurons counted is shown. All molecular weight markers are in kilodaltons.Source data are provided as a Source Data file.

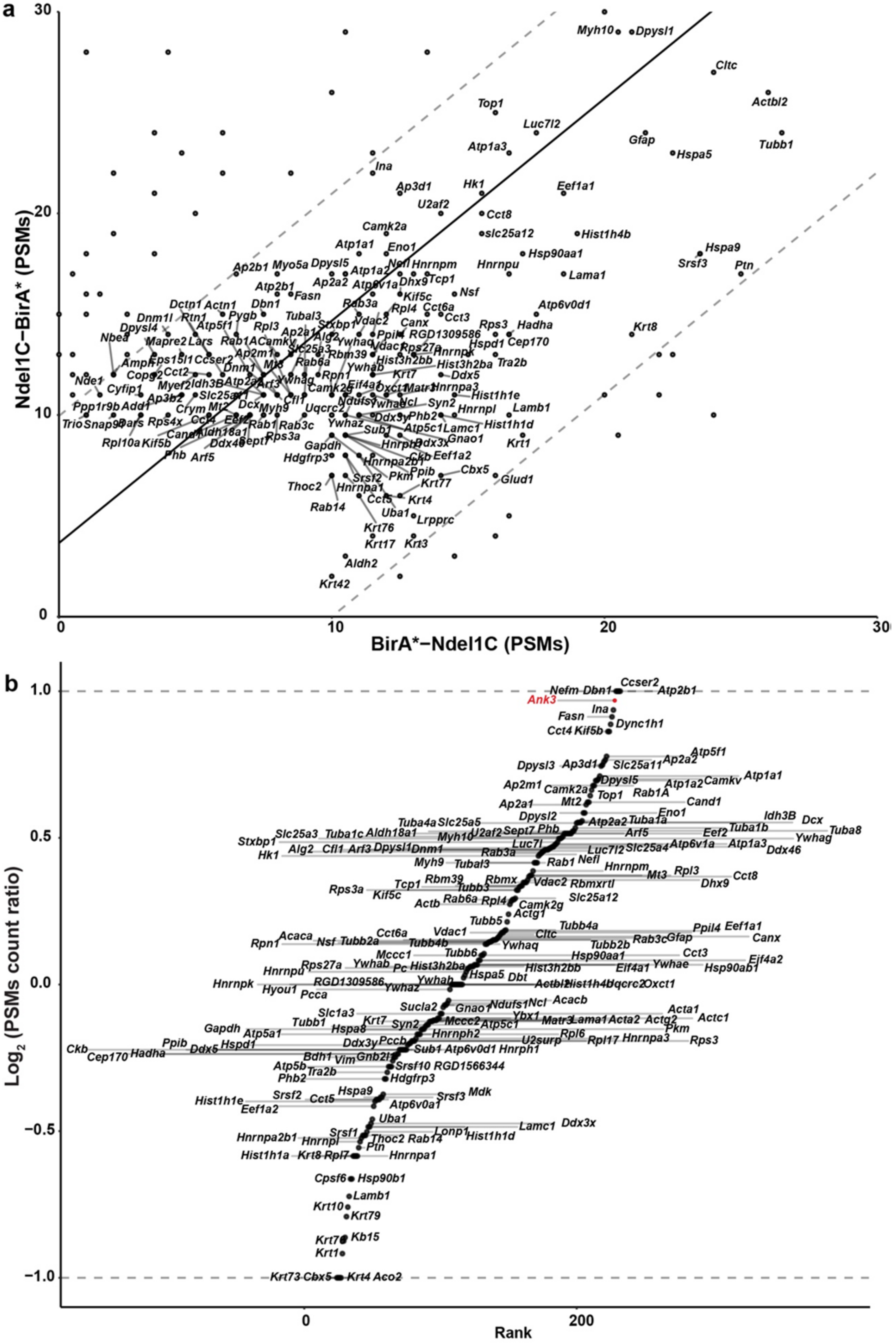

Supplementary Figure 5. Proteins biotinylated equivalently by both Ndel1C-BirA* and BirA*-

Ndel1C. (a) The PSMs for each protein biotinylated by Ndel1C-BirA* and BirA*-Ndel1C, and identified by mass spectrometry. A cutoff of 10 PSMs was used. (b) Rank plot showing the ratio of PSMs for a given protein in the Ndel1C-BirA* samples and the BirA*-Ndel1C samples, i.e. Log_2 (Ndel1C-BirA* PSMs/BirA*-Ndel1C PSMs). Known AIS proteins are shown in red. In (a) and (b), N= 1 for each condition.

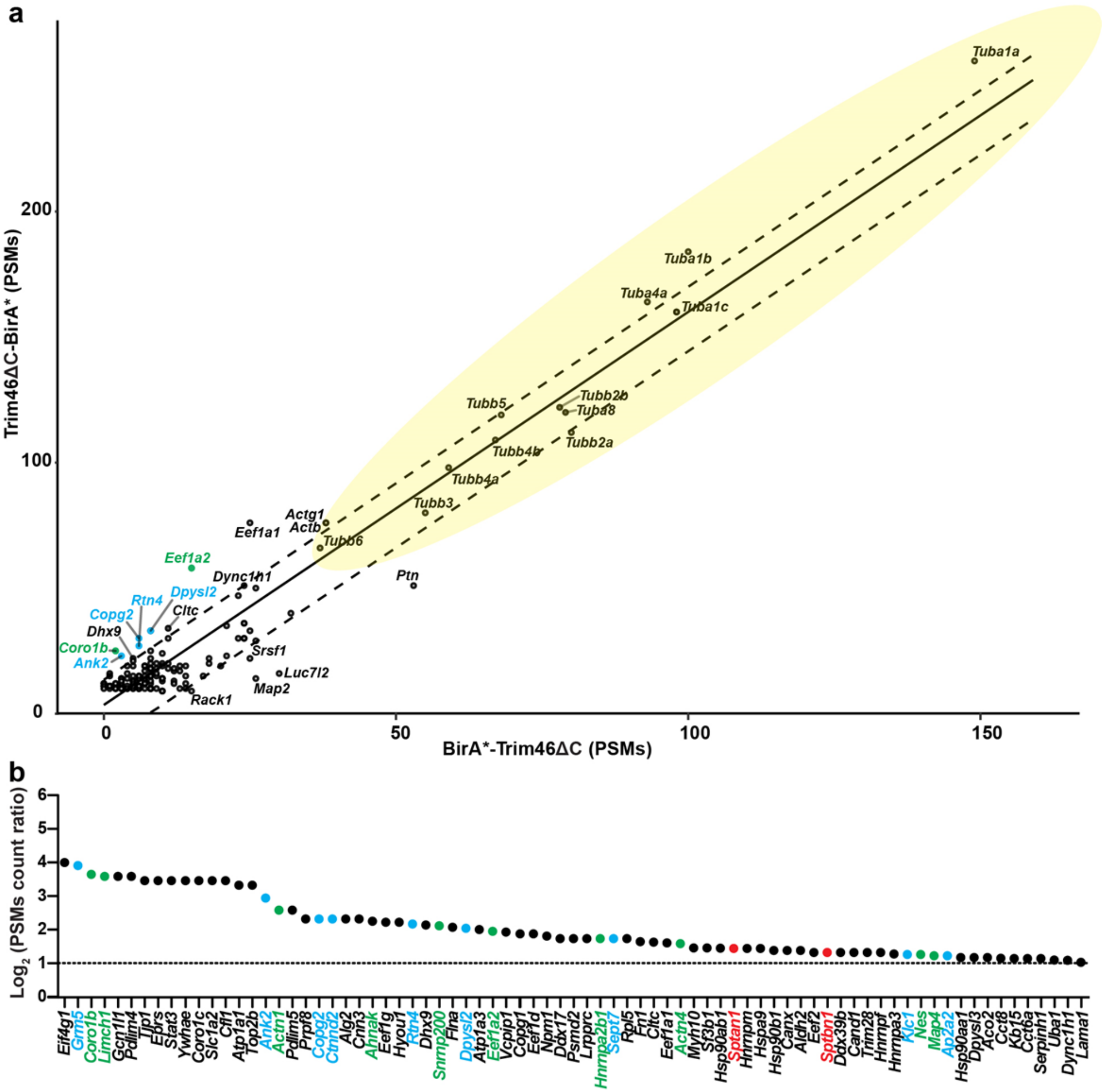

Supplementary Figure 6. Proximity biotinylation using Trim46 chimeras. (a) The number of peptide spectral matches (PSMs) for each biotinylated protein identified by mass spectrometry. The solid line is the least-squares fit of the biotinylated proteins while the dotted lines parallel to the least-squares fit line represent an arbitrary minimum of 10 PSMs confidence interval. Endogenously biotinylated carboxylases have been removed, while tubulins and actin are highly biotinyalted in both Trim46ΔC-BirA* and BirA*-Trim46ΔC -expressing neurons. For clarity and presentation, not all gene names are shown on the graph. (b)The ratio (Log_2) of PSMs Trim46ΔC-BirA* and BirA*-Trim46ΔC -expressing neurons. Known AIS proteins are shown in red. Proteins also identified using NF186-BirA* chimeras are shown in blue, while proteins also

identified using Ndel1C-BirA* chimeras are shown in green. The dashed line indicates two-fold enrichment, or equal levels of biotinylation. N=1 independent experiment.

Supplementary Methods

Primers used in this study.

The pENTR11 shuttle vector was modified by adding the human neuron specific enolase promoter (hENO2) from Addgene (Plasmid #11606). The hENO2 promoter was amplified by PCR and inserted into pENTR11 and named pENTR11-hENO2-MCS.

F-hEno2
GCTAGCGTATGCAGCTGGACCTAGGAGAGAAGCAG
R-hEno2
AGATCTCGGTGGTAGTGGCGGTGGCGGTGGCGGTGG

BirA* was amplified by PCR from pcDNA3.1 Myc-BirA(R118G)-MCS (Addgene, Plasmid #35700) to generate a shuttle template named pENTR11-hENO2-MCS-BirA*-MCS.

F-BirA
GTCGACATGGAACAAAAACTCATCTCAGAAGAG
R-BirA
GCGGCCGCCTTCTCTGCGCTTCTCAGGG

To generate pENTR11-hENO2-HA-NF186-Myc-BirA* and pENTR11-hENO2-HA-NF186ΔFIGQY-Myc-BirA*, cDNA encoding HA-NF186 and NF186ΔFIGQY were amplified by PCR from Rat NF186 tagged with an N-terminal HA epitope tag 53

F-NF186
5'- TTAA CTCGAG GC CAG GCA GCA GGC GCC A -3'
R-NF186
5'- TGAC AAGCTT GG CCA GGG AAT AGA TGG CA -3'
R-NF186deltaFIGQY
5'- TTGC AAGCTT GC CCA AGG GGA CAT CCT T -3'

pENTR11-hENO2-Ndel1C-Myc-BirA* and pENTR11-hENO2-Myc-BirA*-Ndel1C were generated by amplifying Ndel1C (Origene) and inserting it into shuttle template pENTR11-hENO2-MCS-BirA*-MCS. pENTR11-hENO2-Trim46ΔC -Myc-BirA* and pENTR11-hENO2-Myc-BirA*- Trim46ΔC were generated by amplifying Trim46 (Origene) and inserting it into shuttle template pENTR11-hENO2-MCS-BirA*-MCS

F-Ndel1
GCGATCGCCATGGATGGTGAAGATA
R-Ndel1
ATGTCGAGCGGCCGCGTACGCGTCACACTG
F-Trim46
5'- GTAC CTCGAG AA GAA CAT GGA GAA GGA ACT GCT G -3'
R-Trim46
5'- TTAA AGATCT TG ACG GGC GCC TCG GGC ACT -3'
