# Supplementary material for: Mapping axon initial segment structure and function by multiplexed proximity biotinylation
Source: Nat Commun. 2020 Jan 3;11:100. doi: 10.1038/s41467-019-13658-5 (PMC6941957; doi:10.1038/s41467-019-13658-5)
Supplement: Supplementary file 2 — Description of Additional Supplementary Files [file 41467_2019_13658_MOESM2_ESM.pdf]

Description of Additional Supplementary Files

File Name: Supplementary Data 1
Description: This excel file contains all peptide spectral mass counts in the experiments reported here.
